# *N*‐Aryl Acridone Derivatives as Effective Catalysts for Energy Transfer Reactions

**DOI:** 10.1002/chem.71143

**Published:** 2026-05-20

**Authors:** Francesco Calogero, Gaetano D'Onghia, Federica Fina, Zakaria Ziani, Luca Sensoli, Andrea Gualandi, Andrea Fermi, Paola Ceroni, Pier Giorgio Cozzi

**Affiliations:** ^1^ Department of Chemistry “G. Ciamician” ALMA MATER STUDIORUM – Università di Bologna Bologna Italy; ^2^ Center for Chemical Catalysis – C3 ALMA MATER STUDIORUM – Università di Bologna Bologna Italy

**Keywords:** [2+2] reactions, acridones, coumarins, EnT, indoles, photocatalysis

## Abstract

Organic photocatalysts capable of promoting high‐energy triplet energy transfer (EnT) remain limited, particularly for demanding [2+2] photocycloaddition reactions. In this study, we report the use of readily accessible *N*‐aryl acridones as efficient visible‐light‐driven organic photocatalysts for challenging EnT processes. Although largely unexplored in photocatalysis, acridone derivatives exhibit photophysical properties suitable for sensitizing substrates with high triplet energies. Using *N*‐Boc‐indole‐2‐methyl ester and coumarin as representative high‐triplet‐energy substrates, we demonstrate that *N*‐aryl acridones effectively mediate [2+2] cycloadditions under mild conditions via an energy‐transfer mechanism. The modular acridone scaffold enables straightforward structural modification through N‐functionalization, allowing systematic tuning of electronic and steric parameters to optimize catalytic performance.

## Graphical abstract

Acridones derivatives have high triplet energy, are simple accessible, and could be used in challenging [2+2] ENT reactions.

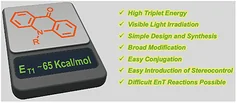

## 1 Introduction

The chemistry of excited (triplet) states of organic molecules exhibits unique reactivity modes, due to their changes in polarizability, bond strengths, and spin density compared to the ground state [1]. The reactivity of excited states was exploited to enable a variety of different synthetic transformations, among them pericyclic reactions [2], atom abstraction reactions [3], or isomerization processes [4].

Direct excitation with ultraviolet (UV) light has long been a unique way to access organic molecules in their excited state and promote unconventional reactivity. The access to triplet state was principally investigated by photoexcitation of the organic chromophore to singlet (S_1_) excited state, followed by intersystem crossing (ISC) to populate T_1_ state, an approach that required harsh conditions by UV‐light irradiation. The process is energetically demanding, as a significant fraction of the input energy is dissipated during population of the excited triplet T_1_ state. Furthermore, for many organic compounds, access to the excited singlet S_1_ state requires high‐energy UV radiation. This introduces important challenges, including selective chromophore excitation, control over the specific electronic states involved, and preservation of molecular stability. Moreover, the use of UV radiation is frequently associated with photodegradation phenomena, since high‐energy photons can induce chemical bond cleavage and compromise sample integrity. Although UV‐absorption spectroscopy is a powerful tool for molecular identification, irradiation often promotes undesirable photochemical side reactions, such as photodimerization or oxidative processes. In addition, the applicability of UV excitation is generally limited to sufficiently conjugated systems and raises concerns related to safety, environmental impact, and long‐term sample stability. Alternative strategies have been investigated at the same time by taking advantage of sensitized population of T_1_ state via energy transfer (EnT) from a catalyst [1, 2, 3, 4]. Energy transfer is formally defined as ‘the photophysical process in which an excited state of one molecular entity (the donor, D) is deactivated to a lower‐lying state by transferring energy to a second molecular entity (the acceptor, A), which is thereby raised to a higher energy state’ [5].

**FIGURE 1:**
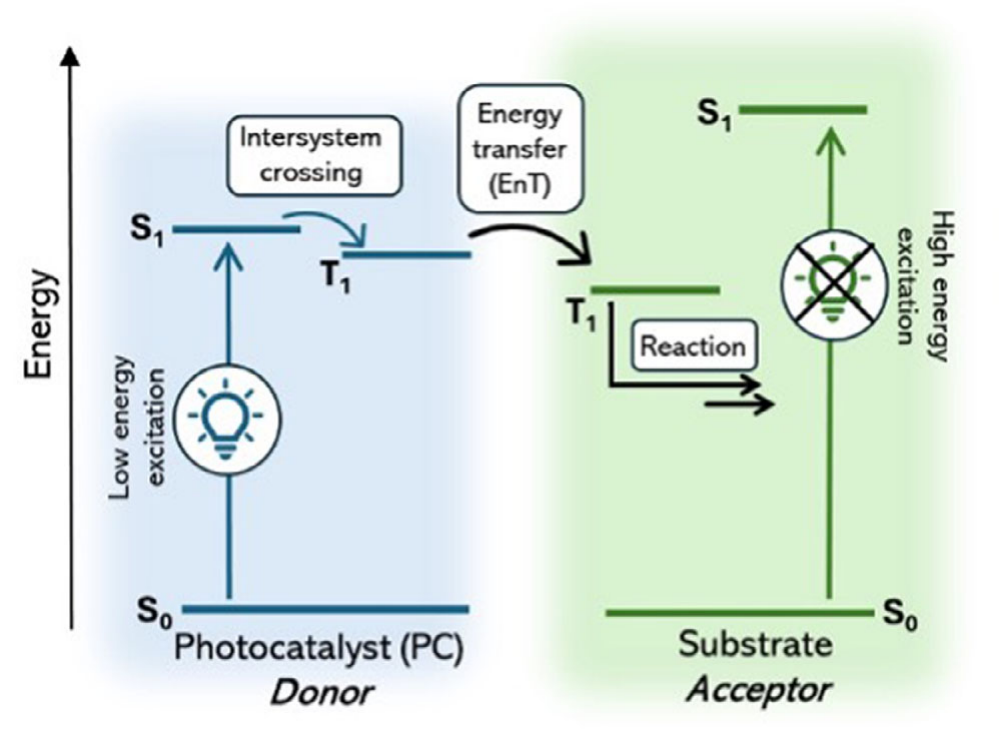
Schematic representation of sensitized population of the organic substrate by selective excitation of the photocatalyst.

The working principle of energy transfer catalysis is based on a photocatalyst (PC) with the following photophysical properties: (i) small energy difference between S_1_ and T_1_ excited states; (ii) very efficient intersystem crossing process S_1_ → T_1_; (iii) sufficiently long lifetime of T_1_ to be engaged in bimolecular energy transfer processes; (iv) proper arrangement of S_1_ and T_1_ excited states of the photocatalyst (the donor, D) and the organic substrate (the acceptor, A) as reported in Figure 1, so that selective excitation of the photocatalyst results into the sensitized population of the organic substrate T_1_ excited state.

**FIGURE 2:**
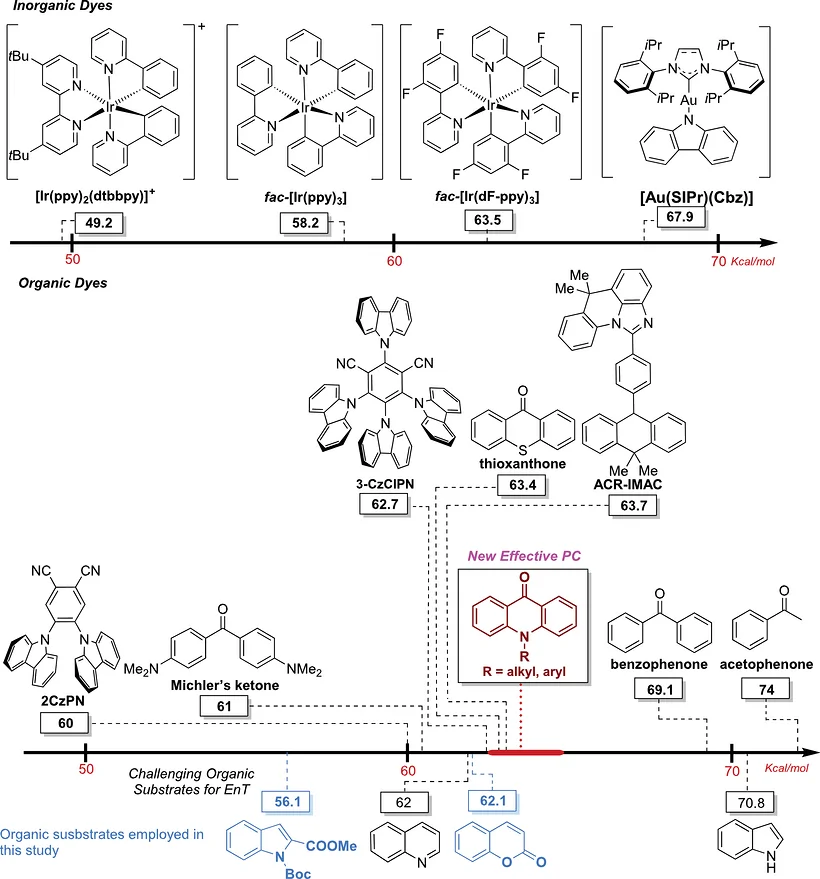
Various photocatalysts for EnT process and the new family of acridone derivatives. Triplet state energies for challenging organic substrates. Data for triplet excited states are reported in kcal/mol.

Several organic and inorganic molecules [6] have been proposed as PCs for EnT reactions (Figure 2). Although iridium complexes have found many applications in these reactions [6], recently Nolan introduced NHC‐gold carbazolyl sensitizers with a high triplet energy. These sensitizers, under 365 nm irradiation, have been shown to promote reactions that were previously inaccessible using iridium‐based catalysts [7]. Figure 2 reports simple organic photocatalysts, particularly aromatic ketones, and their T_1_ energy. In contrast to iridium or gold complexes they are cheap, easily available in large scale, and further their properties are tunable by inserting different functional groups. Moreover, their simple incorporation into chiral moieties for stereoselective photocatalysis is possible, and was already exploited [8]. However, with aromatic ketones side reactions were resulted from direct substrate excitation. Thioxanthone derivatives were successfully used in different reactions [9] both for the absorption close to the visible region (400 nm) [10] and for the high T_1_ energy. It is considered the preferred organic photocatalyst for demanding transformations requiring T_1_ energies > 60 kcal/mol. However, the design of photocatalysts that combine the possibility of visible light excitation (*λ* > 400 nm) and high energy of the T_1_ excited state (> 60 kcal/mol) is highly desirable in difficult [2 + 2] reactions.

In this study, we introduce acridone derivatives in the arena of EnT promoted reactions and for the construction of more sophisticated dual catalysts. Acridone derivatives were studied and characterized from the photochemical point of view [11] and used to access acridinium dyes [12]. Although acridones have been used in photocatalysis, for example by Barham [13], who employed electron‐poor acridones as super‐oxidants, and by Sartorel [14], who used quinacridone‐sensitized photoanodes for C–H activation, to our knowledge, their use in EnT reactions has not been reported before. Acridone derivatives are fully accessible, with the possibility to tune their properties through tailored transformations, and the opened new class of effective and selective EnT transfer catalysts seems promising. In our preliminary studies on this class of compounds, we selected examples of the challenging [2 + 2] reaction of coumarin and indole derivatives with alkenes. We successfully applied an aryl acridone derivative to the EnT reactions and showed that this class of compounds could also be considered for further advances in EnT reactions.

## 2 Results and Discussion

Photocatalytic [2 + 2] cycloadditions promoted by visible‐light in the presence of a photosensitizer were introduced in organic synthesis by Kutal [15], Xiao [16], and Yoon [17], as facile method of constructing cyclobutane rings. The photoreaction with indoles to build cyclobutaneindolines fused bicyclic scaffolds have strong potential in drug discovery. Remarkable examples of intramolecular [2 + 2] cycloaddition of indole with tethered alkenes in the presence of a photocatalys (PC), leading to a functional dearomatization, were reported by You [18], while the intermolecular reaction was just recently explored and is still underdeveloped [19]. In both cases an [Ir(III)] complex was employed as the photocatalyst. Organic photocatalysts [20] usually employed for investigating this type of reactions, such as rose Bengal [21], Michler's ketone [22], and riboflavin [23] were unsuccessful, all giving no reaction (0% yield). Thioxantone [10], a powerful organocatalyst employed in EnT reaction [24, 25, 26] was not investigated in this transformation. Organic dyes displaying thermally activated delayed fluorescence (TADF) [27] such as 1,2‐bis(carbazol‐9‐yl)‐4,5‐dicyanobenzene (2CzPN) [28], were quite useful only in intramolecular cyclizations. Other TADFs dyes such as 3DPA2FBN, 4CzIPN, and 3CzClIPN [27] were also found able to promote intramolecular [2 + 2] reactions but in various yields, while the TADF material ACR‐IMAC (imidazoacridine‐based) was reported very effective in dearomative and non‐dearomative intra‐ and intermolecular [2 + 2] cycloadditions, due to their triplet energy (*E*_T1_ = 58.9–69.9 kcal/mol) [29]. In addition, 4CzIPN [30] was found capable of promoting a [2+2] cycloaddition of electron‐deficient styrenes forming the corresponding cyclobutane products bearing electron‐deficient aryl substituents [31]. More recently, the [2+2] cycloaddition of coumarins with diverse olefins under visible‐light irradiation was reported with the organic photocatalyst 4CzIPN [32], using dimethyl carbonate as green reaction solvent, under irradiation with a 25 W blue LED. Interestingly the strategy was implemented toward the synthesis of (±)‐lindleyanin, a natural occurring fused system containing a cyclobutene moiety.

Furthermore, as the energy transfer process [33] is endergonic for most of the PCs, the introduction of carboxylic group in position 2 of the indole substrate lowers the energy of its T_1_ state to ca. 60 kcal/mol, making the corresponding energy transfer process exergonic [22]. It is also important to select an adequate protecting group for the nitrogen atom of the indole. Unprotected indole‐2‐carboxylic esters and *N*‐benzyl‐protected analogues were reported unreactive substrates under EnT reaction with alkenes, although their evaluated E_T1_ energy was lower (57 kcal/mol) than that of the *N*‐Boc derivatives [19]. In fact when we used the unprotected **1a** no reaction was observed. Therefore, *N*‐Boc indole‐2‐methyl ester **1a** was the selected substrate to explore the acridone derivatives in EnT reactions. Acridone derivatives are well‐known and have been reported extensively [34], and among the aryl ketones, are able to promote photochemical reactions [35, 36].

Unsubstituted acridone (9(10*H*)‐acridone) was synthesized as early as 1912 [37] and its preparation was described in an Organic Synthesis in 1939 [38]. Since then, additional methodologies have been developed to diversify the range of acridones that can be produced through organic synthesis, including Friedel–Crafts chemistry [39] or iterative nucleophilic aromatic substitution (S_N_Ar) reaction [40]. These methodologies were also applied to the synthesis of acridone alkaloids [41]. Acridone possesses strong fluorescence emission [42] and has been used as the chromophore in fluorescent chemosensors [43] or incorporated in cages [44]. The photodimerization of acridone in the presence of quenchers was also reported [45]. The variability, synthetic accessibility, and ability to introduce a functional group make this scaffold unique compared to other ketones used as photocatalyst. Furthermore, the NH group is ideal for attaching different groups or chains bearing other functionalities. However, since there are no studies on even simple acridone derivatives promoting EnT reactions, we decided to start with some very simple acridone derivatives for our preliminary investigation.

The acridones **I–IV** derivatives were prepared by straightforward reactions [46, 47] (see Supporting Information for details), using copper promoted cross‐coupling reaction or direct alkylation of the acridone anion. They were employed as photocatalyst with **1a** in the presence of the vinylsilane **2a**. In Table 1 the salient data about the reaction optimization are reported. As it is possible to evince by the results, aryl and alkyl substituted acridones can promote the reaction, and the efficiency is controlled by electronic effects on the aryl substituent, with the 3,5‐(CF_3_)_2_C_6_H_3_‐ substituent giving the higher yields (Table 1, entry 1) [43]. The photocatalyst and light are essential for the reaction (Table 1, entries 2 and 3). Differently from thioxanthones and other organic dyes used for EnT reactions, 427 irradiation is sufficient because the absorption onset of the photocatalysts is superimposed to the LED profile at lower wavelengths (Figure S6). The reaction works in DMF, MeCN and MeOH as solvent, while DCM and THF are giving just modest conversion (Table 1, entries 12–15). This is imputable to the low solubility of acridone derivatives in DCM and THF. For all reactions a good d.r. in favor for the *syn*‐diastereoisomer was observed, as previously observed with [Ir(III)] photocatalyst [19]. As thioxantone is a well‐known, commercially available, and effective catalyst for [2+2] additions [10], so we compared its performance under our reaction conditions (Table 1, entry 11) and found slightly reduced reactivity.

**TABLE 1:** Reaction optimization using Boc‐indole **1a** and vinylsilane **2a**.

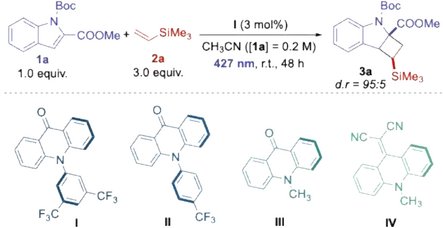		
Entries^a^	Deviation from standard conditions	Yield (%)^b^
**1**	**None**	**75**
2	No light^c^	No reaction
3	Kessil PR160L@456 nm instead of @427 nm	Traces
4	No photocatalyst	No reaction
5	Reaction performed under air	Traces
6	24 h Irradiation time	66^d^
7	5 mol% of **I**	76
8	**II** Instead of **I** ^e^	45^d^
9	**III** Instead of **I** ^e^	64^d^
10	**IV** Instead of **I** ^e^	Traces
11^f^	Thioxanthone instead of **I**	65
12	MeOH instead of CH_3_CN	61^d^
13	DMF instead of CH_3_CN	65^d^
14	THF instead of CH_3_CN	27^d^
15	DCM instead of CH_3_CN	12^d^

^a^ Reaction performed on 0.1 mmol scale. D.r. was evaluated by ^1^H‐NMR analysis of the reaction crude by comparison of the protons in the benzylic position. For all the entries 95:5 *syn:anti* ratio was observed.

^b^ Isolated yield after chromatographic purification.

^c^ 48 h reaction time.

^d^ Evaluated by ^1^H‐NMR analysis with the use of internal standard (1 equivalent of benzyl alcohol was added as internal standard).

^e^ 5 mol% of photocatalyst.

^f^ The reaction was carried out using commercially available thioxanthone in the optimized reaction conditions at 427 nm.

**SCHEME 1:**
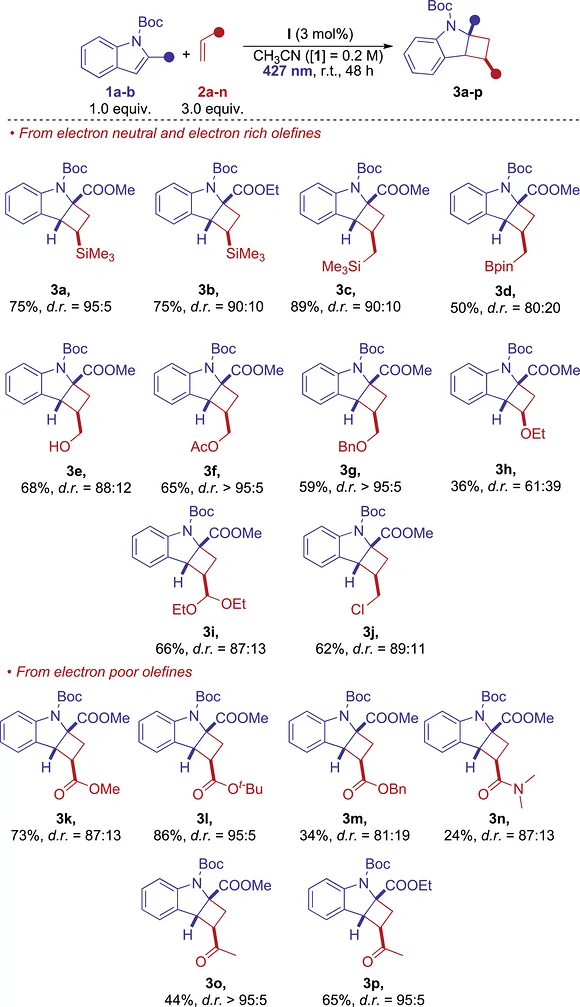
Examples of [2 + 2] reaction of *N*‐Boc indoles with selected alkenes promoted by the catalyst **I**.

With the optimal catalyst and reaction conditions, we evaluated the reactivity of **1a** toward a range of substituted alkenes. To study the scope, we used different alkenes (electron rich or electron poor) as shown in Scheme 1, operating with a blue‐LEDs at 427 nm, in MeCN. In general, a good *syn*:*anti* ratio was observed with electron‐rich and electron‐poor alkenes, with the *syn* diastereoisomer predominantly formed in all reactions.

The diastereoselectivity is maintained in the range of 61:39 to 95:5, while the isolated yields are generally good, with a few electron‐poor alkenes (amide, ketone and benzylic ester) that afforded the desired compound in reduced yields. The methodology is giving similar results to those obtained with a more expensive iridium catalysts [21].

Since chromanones are privileged scaffolds found in many natural products [48], pharmaceutical compounds [48], flavors and fragrances [49], we explored the synthesis of cyclobutane‐condensed chromanones using our new acridone photocatalysts. Due to the high triplet energy of coumarin (*E*_T1_ = 62.4 kcal/mol for **1c**) coumarins are rarely studied in EnT. The [2 + 2] photodimerization of coumarins was reported by irradiation with high pressure mercury lamps in 1969 [50]. Lewis acid can promote the intramolecular [2 + 2] cycloaddition by lowering the triplet energy as reported by Bach [51] in a stereoselective manner. In addition, Iridium(II) complexes [52], thioxanthone [53], and gold complexes [7] were reported to be active catalysts in the [2 + 2] cycloaddition of coumarins with alkenes.

**SCHEME 2:**
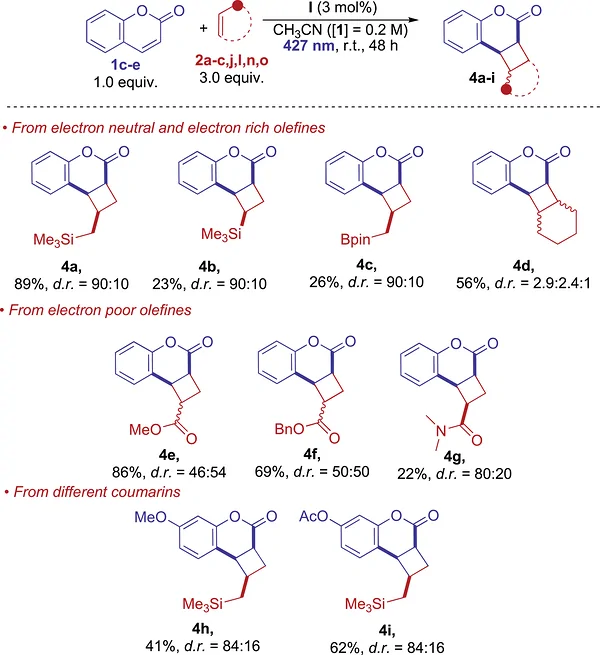
Examples of [2 + 2] reaction of coumarins promoted by acridone catalyst **I** at 427 nm.

We wondered whether the simple prepared catalyst **I** would also be suitable for the challenging reaction of coumarins and alkene. The acridone catalyst **I** could induce the [2 + 2] intermolecular reactions between various alkenes and coumarins, as shown in Scheme 2.

Although the reaction is slow compared to the one conducted in the presence of [Au(SIPr)(Cbz)] (SIPr = [*N*,*N*‐bis(2,6‐diisopropylphenyl)imidazolin‐2‐ylidene]; Cbz = carbazolyl) catalyst recently reported by Nolan [7], we carried out the [2 + 2] reaction employed LED peaked in the visible region of the spectrum (427 nm). The reaction conditions from Scheme 1 were directly applied without (further) optimization, with an excess of alkene (3 equiv.) and irradiation in the presence of a sensitizer, leads to formation of the anti‐head‐to‐head dimer as the predominant isomer in moderate to good yields, depending on the alkene employed. While vinyltrimethylsilane afforded product **4b** as a single regioisomer, in high diastereomeric ratio (90:10) and in an 23% isolated yield, allylsilane was found much more reactive (89% isolated yields, **4a**). In general, formation of the same regioisomer was observed with all examined terminal alkenes. Although boron substituted alkene was found compatible with the reaction conditions, isolated yields of **4c** were modest.

Reaction with an internal alkene, cyclohexene, led to a mixture of three diastereomers of **4d** (2.9:2.4:1, in order of relative abundance) in 56% overall yield. By careful chromatographic separation we were able to isolate a single diastereoisomer and assign its configuration (see Supporting Information) for details. Recently, the [2+2] cycloaddition of coumarin with an alkene was described using two types of organic dyes [32, 54] and our yields, diastereoselective and conditions are comparable to those reported using 5 mol% of the 4CzIPN catalyst [32]. The photocatalyst 3‐thiophenyl‐4‐hydroxycoumarin recently reported by Melchiorre [54] produced a similar diastereoisomeric ratio for **4d**.

Acrylates, which are considered activated alkenes in [2 + 2] cycloadditions, were found to be reactive with the system, exhibiting diastereoselectivity independent of ester group hindrance (products **4e** and **4f**). In one case (**4f**) we were able to isolate a single diastereoisomer by chromatographic purification. We observed that the reaction of coumarin with acrylate was not diastereoselective. With 4CzIPN hindered *t*‐butyl acrylate exhibited similar behavior. Two differently substituted coumarins (with OMe and OAc groups) were also evaluated in a reaction with allyl silane [32]. This reaction produced the desired product with moderate yield and high d.r. For these products, during the chromatographic purification the ratio of diastereoisomer obtained was slightly altered toward the most abundant diastereoisomer (94:6 for **4h**; 90:10 for **4i**), differently from the other cases. Finally, also unsaturated amide gave the product **4g**, but in low yields, with a 4:1 diastereoselectivity.

**SCHEME 3:**
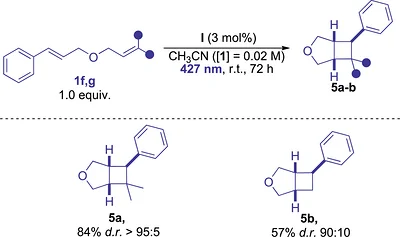
Examples of intramolecular [2+2] cycloaddition employing the catalyst **I** at 427 nm.

Styrene derivatives can undergo sensitized photochemistry from an easily accessible triplet. Intramolecular reaction of cinnamyl derivatives were reported by Yoon using white light (23 W) in the presence of 1 mol% {Ir[dF(CF_3_)ppy]_2_(dtbpy)}PF_6_ catalyst [55]. Recently, Booker‐Milbur and coworker, by TD‐DFT calculation have design a large family of thioxanthone derivatives that span the UV−visible (vis) region (300−450 nm) [9]. Based on this design, a methoxy derivative of thioxanthone was able to promote the intramolecular [2 + 2] cycloaddition operating at 455 nm, in few hours. The advantage to use organic photosensitizers [56, 57] for the intramolecular [2 + 2] cycloaddition prompted us to investigate this model reaction with two substrates, simply obtained from cinnamyl alcohols by alkylation with allyl and methallyl bromides (see Supporting Information for details about the preparation). The reaction, in both cases gave the desired compounds with high diastereoselectivity, and from good to excellent yields, showing that our catalyst could be also applicable to intramolecular reactions (Scheme 3).

### 2.1 Photophysical Studies

**FIGURE 3:**
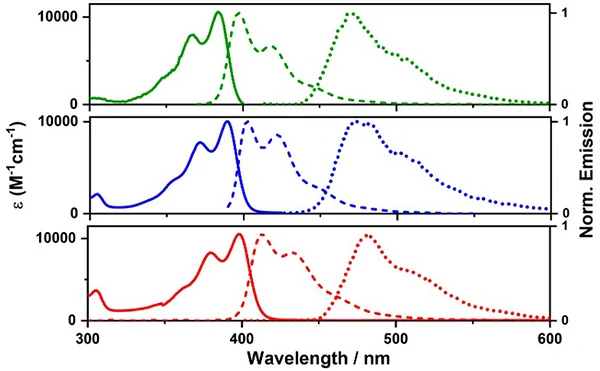
Absorption (solid lines) and steady state fluorescence (dashed lines) at room temperature in acetonitrile; phosphorescence in rigid matrix of butyronitrile at 77K (dotted lines) of photocatalyst **I** (green lines), **II** (blue lines), and **III** (red lines). *λ*_ex_ = 390 nm.

Acridones **I**–**III** exhibit similar UV–vis absorption and fluorescence spectra, with onset of absorption in the range 400–415 nm and fluorescence maxima around 405 nm (Figure 3; Figures S3, S5, and S6), in agreement with published results [58].

More remarkable differences are observed in fluorescence quantum yields (Table 2) and non‐radiative rate constants (k_nr_) of deactivation of the fluorescent excited state. Acridones **I** and **II** possess higher values of *k*_nr_: this could be related to a higher rate constant of ISC between S_1_ and T_1_ excited states—a desirable property for a triplet photosensitizer, as a larger fraction of excited S_1_ states is converted into the reactive T_1_ state.

**TABLE 2:** Photophysical properties of acridones **I**–**III** in air‐equilibrated acetonitrile solution, unless otherwise noted.

Acridones	*τ*/ns	*Φ*_fluo_	*k*_fluo_/s^−1^	*k*_nr_/s^−1^	*E*_T1_(Kcal/mol)^a^
**I**	1.1	0.40	4.0 × 10^8^	6.0 × 10^8^	64.6
**II**	1.9	0.21	2.1 × 10^8^	7.9 × 10^8^	63.1
**III**	6.4	0.60	1.6 × 10^8^	1.0 × 10^8^	62.9

^[a]^ ET1 values were determined considering the wavelength corresponding to 10% of phosphorescence maximum. In butyronitrile rigid matrix at 77 K.

To confirm whether an energy transfer mechanism is possible, the energy of the lowest‐lying triplet state was obtained for acridones **I**–**III** by recording phosphorescence spectra in rigid matrix at 77 K (Figure 3; Figures S3, S5, and S6). The T_1_ energy values are comparable and in the range between 62 and 65 kcal/mol, with higher values for acridones featuring an electron poor *N*‐substituted arene. Preliminary quenching studies (Figure S8) allowed us to exclude that vinyltrimethylsilane could act as a quencher for the acridones excited T_1_ state, as expected by the highly endergonic nature of the corresponding energy transfer process. On the other hand, triplet‐triplet energy transfer process from photocatalysts **I**–**III** to coumarin [59] and indole substrates is thermodynamically allowed: for example, compounds **1a** and **1c**, selected as reference compounds, display triplet energy of 60.0 [22], and 62.4 kcal/mol [7], respectively.

To evaluate this process, a Stern‐Volmer analysis was performed (Figures S9–S11). The study was carried out on compound **I**, which displayed the best experimental results (Table 3), and on the less performing acridone **III**, for comparison. For the quenching experiment, the decay of the characteristic absorption band of T_1_ state in the range 580 – 600 nm was recorded by transient absorption spectroscopy upon laser excitation at 355 nm (Figures S9 and S11) since no phosphorescence is observed from the T_1_ state of acridones in degassed solution at room temperature.

**TABLE 3:** Photophysical properties of acridones **I** and **III** in air‐equilibrated acetonitrile solution, unless otherwise noted.

Acridones	Quencher	*k*_q_/M^−1^s^−1^
**I**	Indole, **1a**	1.5 × 10^9^
	Coumarin, **1c**	4.6 × 10^9^
**III**	Indole, **1a**	6.6 × 10^8^
	Coumarin, **1c**	9.2 × 10^8^

As shown in Table 3, compound **I** features quenching constants one order of magnitude higher than those observed for compound **III** in the presence of both reaction substrates. This aligns with the respective triplet energy values. The favorable photophysical features of acridone **I** and the highest quenching constants for coumarin and indole are consistent with the highest reaction yields obtained for this photocatalyst. To confirm that only the excited T_1_ state of the photocatalysts is involved in the reaction mechanism, quenching experiments on the fluorescence time decay were performed, to probe the decay of the short‐lived S_1_ state. Using quencher concentrations comparable to those used in the reactions (Table 1 and Scheme 1), no change in fluorescence decay was observed. This result highlights the importance of selecting photocatalysts in which a high fraction of the excited state populated by absorption of light (S_1_ or higher singlet states) efficiently converts into the reactive triplet (T_1_) state, which must also possess sufficient energy to enable the desired transformation.

## 3 Conclusion

Cheap and affordable *N*‐aryl acridones were found to be quite effective in intermolecular [2 + 2] cycloaddition of alkene to indoles and coumarins. Compound **I** and other acridone derivatives are suitable photocatalysts for EnT reactions, overcoming the use of expensive metal iridium or gold catalysts. These catalysts are easily accessible by copper cross‐coupling reactions or simple alkylation. Unlike other known organic catalysts such as thioxantone, the use of a 427 nm LED is sufficient to induce the desired reaction. The triplet energy achieved by catalyst **I** (64.6 kcal/mol) was found to be effective in promoting the reaction with a class of difficult substrates such as indole derivatives and coumarin with moderate to good results. The possibility of decorating acridone or modifying the structure of acridone by simple chemistry makes these compounds attractive for further studies and developments, including stereoselective variants of [2 + 2] reactions. More importantly, by design, it would be possible to increase the triplet energy and explore this new class of compounds in even more challenging substrates, such as simple ketones for the Paternò‐Buchi reactions. Studies along these lines are underway in our laboratories.

## Conflicts of Interest

The authors declare no conflicts of interest.

## Supporting information



The authors have cited additional references within the Supporting Information [32, 46, 60, 61, 62, 63, 64, 65, 66, 67, 68, 69, 70].
**Supporting file**: chem71143‐sup‐0001‐SuppMat.docx.

## Data Availability

The data that supports the findings of this study are available in the Supporting Information of this article.
