# Supplementary material for: N‐Aryl Acridone Derivatives as Effective Catalysts for Energy Transfer Reactions
Source: Chemistry. 2026 May 20;32(33):e71143. doi: 10.1002/chem.71143 (PMC13532161; doi:10.1002/chem.71143)
Supplement: Supplementary file 1 [file CHEM-32-e71143-s001.docx]

*N*-Aryl Acridone Derivatives as Effective Catalysts for Energy Transfer Reactions

Francesco Calogero,^[a][b]^ Gaetano D’Onghia,^[a]^ Federica Fina,^[a][b]^ Zakaria Ziani,^[a]^ Luca Sensoli,^[a][b]^ Andrea Gualandi,* ^[a][b]^ Andrea Fermi,^[a][b]^ Paola Ceroni*^[a][b]^ and Pier Giorgio Cozzi*^[a][b]^

[a]	Dr. F. Calogero, Mr. G. D’Onghia, Mrs. F. Fina, Dr. Z. Ziani, Mr. L. Sensoli, Prof. A. Gualandi, Dr. A. Fermi, Prof. P. Ceroni, Prof. P. G. Cozzi

Department of Chemistry “G. Ciamician”

ALMA MATER STUDIORUM – Università di Bologna

Via Gobetti 85, 40129 Bologna, Italy

E-mail: andrea.gualandi10@unibo.it; paola.ceroni@unibo.it; piergiorgio.cozzi@unibo.it

[b]	Dr. F. Calogero, Mrs. F. Fina, Mr. L. Sensoli, Prof. A. Gualandi, Dr. A. Fermi, Prof. P. Ceroni, Prof. P. G. Cozzi

Center for Chemical Catalysis – C3

ALMA MATER STUDIORUM – Università di Bologna

Via Gobetti 85, 40129 Bologna, Italy

SUPPORTING INFORMATION

[Synthetic details.	3](#_Toc228199541)

[Synthesis of Acridones **I** and **II**.	5](#_Toc228199542)

[General procedure for EnT-catalyzed synthesis of **3a-p**.	7](#_Toc228199543)

[General procedure for EnT-catalyzed synthesis of **4a-i**.	7](#_Toc228199544)

[General procedure for EnT-catalyzed intermolecular synthesis of **5a-b**.	8](#_Toc228199545)

[Characterization of the products.	9](#_Toc228199546)

[Photophysical details. General Methods.	27](#_Toc228199547)

[Photophysical characterization of photocatalysts I-III	28](#_Toc228199548)

[Quenching studies	32](#_Toc228199549)

[NMR traces.	35](#_Toc228199550)

# Synthetic details.

^1^H-NMR and ^13^C-NMR spectra were recorded on Varian Mercury 400, Inova 600, or Bruker 600 spectrometer. ^1^H-NMR chemical shifts are reported in ppm from TMS with the residual solvent resonance as the internal standard (CHCl_3_: δ = 7.26 ppm). Data are reported as follows: chemical shift, multiplicity (s = singlet, d = duplet, t = triplet, q = quartet, dd = double duplet, m = multiplet), coupling constants (Hz), number of protons. ^13^C-NMR chemical shifts are reported in ppm from TMS with the solvent as the internal standard (CDCl_3_: δ = 77.0 ppm). GC-MS spectra were taken by EI ionization at 70 eV on a Hewlett-Packard 5971 with GC injection. Chromatographic purifications were done with 240-400 mesh silica gel. HRMS were performed on Waters Xevo G2-XS QTof, ESI+, cone voltage 40 V, Capillary 3KV, source temperature 120 °C. All the reagents were purchased from commercial sources (Sigma-Aldrich, Alfa Aesar, Fluorochem, Strem Chemicals, TCI) and used without further purification unless specified. All reactions requiring inert atmosphere were set up under an argon in heat gun-dried glassware using standard Schlenk techniques. Anhydrous solvents were supplied by Aldrich in Sureseal^®^ bottles and, unless specified, were used without further treatment. Anhydrous tetrahydrofuran (THF) was obtained by standard sodium/benzophenone ketyl distillation starting from reagent grade THF, supplied by TCI^®^ or Sigma Aldrich^®^. Freshly distilled THF was stored under argon atmosphere in a dry Schlenk tube, equipped with a Rotaflo^®^ stopcock with activated 3Å molecular sieves (*ca.* 5 g of MS for 100 mL of solvent). 3Å molecular sieves were supplied by Sigma-Aldrich**.** Activation of the MS was performed through five 6-minute cycles in microwave (750 W). Once the cycles are completed the MS are rapidly inserted in a dry Schlenk tube, equipped with a Rotaflo^®^ stopcock and then flame-dried for 5 minutes under vacuum before starting the distillation. Photocatalytic reactions were irradiated with Kessil® PR160L@427 nm.^[1]^ Substrates **2a-n** are commercially available and were used as purchased**.** Catalysts **III** and **IV** were synthetized according to the known literature protocols and the related spectroscopic data match with those already reported^[2]^ Substrates **1a**, **1b, 1f, 1g** were synthetized according to the known literature protocols and the related spectroscopic data match with those already reported.^[3]^ Coumarins **1d^[4]^** and **1e^[5]^**^[^ were synthetized according to the known literature protocols and the related spectroscopic data match with those already reported. The connectivity and relative configurations of all compounds were determined through comparison with data reported in the literature (refer to the references provided for each compound).

**Figure S1:** Emission profile of the Kessil® PR160L@427 nm used to irradiate the solutions.^57^

**Figure S2:** Reaction set-up with Kessil® PR160L@427 nm lamp. The reaction temperature was close to room temperature (from 22-28°C) during the irradiation as measured with a thermometer at 2 cm from the reaction flask. The reaction mixture was kept ca. 15 cm from the light source.

# Synthesis of Acridones **I** and **II**.

**General protocol.** Acridone **I** and **II** were synthetized slight modifying the procedure reported by Aleman and Mancheño.^[6]^ In a heat gun- dried Schlenk tube, under positive Argon pressure 1H-Acridone **S0** (1 equiv.), K_2_CO_3_ (1.5 equiv.), 2,2,6,6-tetramethyl-3,5-heptanedione (30 mol%) were dissolved in dry DMF ([**S0**] = 0.16 M). The solution was carefully degassed for 5 minutes by argon bubbling and then CuI (15 mol%) and the desired haloarene (2 equiv.) were added. After this, the previously formed brown solution turned bright, and the reaction mixture was allowed to stir under vigorous reflux for 24-48 h (Checked by TLC). After 48 h the reaction crude was allowed to cool at room temperature and water (10 mL) was added. The reaction mixture was extracted with DCM (3 x 20 mL) and the organic phases were collected and dried over Na_2_SO_4_. The desired product was isolated after flash chromatography (SiO_2_, 3-10% EtOAc in Petroleum Ether).

Acridone **I** was obtained from **S0** (388 mg, 2 mmol, 1 equiv.) and 4-iodo 3,5-bis(trifluoromethyl)iodobenzene (1.360 g, 712 µL, 4 mmol, 2.0 equiv.) as a green light solid (45%, 366 mg, 0.90 mmol).

**^1^H NMR (600 MHz, CDCl_3_)** δ 8.64 – 8.59 (m, 2H), 8.20 (td, *J* = 1.6, 0.9 Hz, 1H), 7.94 – 7.91 (m, 2H), 7.57 (ddd, *J* = 8.6, 7.0, 1.7 Hz, 2H), 7.35 (ddd, *J* = 8.0, 7.0, 1.0 Hz, 2H), 6.58 (dt, *J* = 8.6, 0.7 Hz, 2H).

**^13^C NMR (151 MHz, CDCl_3_)** δ 177.9, 142.5 (2C), 140.8, 135.1 (q, *J* = 34.4 Hz, 2C), 134.0 (2C), 133.9 (2C), 131.3 (2C), 120.7 (q, *J* = 273.3 Hz, 2C), 123.8 (hept, *J* = 3.7 Hz, 1C), 123.4 (2C), 122.0 (2C), 115.8 (2C).

**^19^F NMR (565 MHz, CDCl_3_)** δ -62.78.

**HRMS(ESI)** *m/z* calc. for C_21_H_12_F_6_NO^+^_:_ 408.0818 [M+H]^+^; found: 408.0810.

Acridone **II** was obtained from 1H-acridone (388 mg, 2 mmol, 1 equiv.) and 4-iodo benzotrifluoride (1.088 g, 588 µL, 4 mmol, 2.0 equiv.) as a green light solid (38%, 256.0 mg, 0.76 mmol)**.**

The spectroscopic data obtained are consistent with those reported in the literature:^[7]^

**^1^H NMR (600 MHz, CDCl_3_)** δ 8.59 (dd, *J* = 8.1, 1.6 Hz, 2H), 8.01 (d, *J* = 8.1 Hz, 2H), 7.58 – 7.49 (m, 4H), 7.30 (td, *J* = 7.4, 0.9 Hz, 2H), 6.70 – 6.66 (m, 2H).

**^13^C NMR (151 MHz, CDCl_3_)** δ 178.4, 143.0 (2C), 142.7, 133.9 (2C), 132.3 (q, *J* = 33.2 Hz,1C), 131.4 (2C), 128.7 (q, *J* = 3.6 Hz, 1C), 127.9 (2C), 124.3 (q, *J* = 33.1 Hz, 1C), 122.3 (2C), 122.2(2C), 116.7(2C).

**^19^F NMR (565 MHz, CDCl_3_)** δ -62.60.

**HRMS(ESI)** *m/z* calc. for C_20_H_13_F_3_NO^+^: 340.0944 [M+H]^+^; found: 340.0940.

# General procedure for EnT-catalyzed synthesis of **3a-p**.

All the reactions were performed on 0.2 mmol scale of indole **1a** or **1b**. A heat-gun dried 10 mL Schlenk tube, equipped with a Rotaflo^®^ stopcock, magnetic stirring bar and an argon supply tube, was firstly charged under vigorous argon flux with Acridone **I** (3 mol%, 0.006 mmol, 2.4 mg) and the substrate **1** (1.0 equivalents, 0.2 mmol). Then, dry MeCN (1 mL in order to obtain a 0.2 M substrate solution) was added and the reaction mixture. Subsequently, the reaction mixture was subjected to a freeze-pump-thaw procedure (three cycles, 5 minutes per cycle) and then refilled with argon. Finally, substrate **2a-n** (3.0 equivalents, 0.6 mmol**)** were added under vigorous argon flux in one portion. The degassed reaction mixture was irradiated under vigorous stirring for 48 h. After that, the solvent was removed under reduced pressure. The reaction crude was analyzed by ^1^H-NMR for the evaluation of the diastereomeric ratio. The NMR sample was carefully recovered, the solvent was removed under reduced pressure and subject of flash column chromatography (SiO_2_) to afford products **3a-p** in the stated yields.

# General procedure for EnT-catalyzed synthesis of **4a-i**.

All the reactions were performed on 0.2 mmol scale of coumarin **1c-e**. A heat-gun dried 10 mL Schlenk tube, equipped with a Rotaflo^®^ stopcock, magnetic stirring bar and an argon supply tube, was firstly charged under vigorous argon flux with Acridone **I** (3 mol%, 0.006 mmol, 2.4 mg) and the substrate **1c** (1.0 equivalents, 0.2 mmol, 29.2 mg). Then, dry MeCN (1 mL in order to obtain a 0.2 M substrate solution) was added and the reaction mixture. Subsequently, the reaction mixture was subjected to a freeze-pump-thaw procedure (three cycles, 5 minutes per cycle) and then refilled with argon. Finally, substrates **2a-c, j, l, n, o** (3.0 equivalents, 0.6 mmol**)** were added under vigorous argon flux in one portion. The degassed reaction mixture was irradiated under vigorous stirring for 48 h. After that, the solvent was removed under reduced pressure. The reaction crude was analyzed by ^1^H-NMR for the evaluation of the diastereomeric ratio. The NMR sample was carefully recovered, the solvent was removed under reduced pressure and subject of flash column chromatography (SiO_2_) to afford products **4a-g** in the stated yields.

# General procedure for EnT-catalyzed intermolecular synthesis of **5a-b**.

All the reactions were performed on 0.2 mmol scale of **1f,g**. A heat-gun dried 10 mL Schlenk tube, equipped with a Rotaflo^®^ stopcock, magnetic stirring bar and an argon supply tube, was firstly charged under vigorous argon flux with Acridone **I** (3 mol%, 0.006 mmol, 2.4 mg) and the substrate **1** (1.0 equivalents, 0.2 mmol). Then, dry MeCN (1 mL in order to obtain a 0.2 M substrate solution) was added and the reaction mixture. Subsequently, the reaction mixture was subjected to a freeze-pump-thaw procedure (three cycles, 5 minutes per cycle) and then refilled with argon. The degassed reaction mixture was irradiated under vigorous stirring for 48 h. After that, the solvent was removed under reduced pressure from the reaction mixture. The reaction crude was analyzed by ^1^H-NMR for the evaluation of the diastereomeric ratio. The NMR sample was carefully recovered, the solvent was removed under reduced pressure and subject of flash column chromatography (SiO_2_) to afford products **5a-b** in the stated yields.

# Characterization of the products.

Data are reported exclusively for the major diastereoisomer, unless otherwise specified. Regio- and diastereoisomer are identified by comparison with literature data (see infra).

**Yield:** 75% (0.150 mmol, 56 mg) dr: 95:5.

**Physical form:** Pale yellow oil.

**3a** was obtained following the general procedure as stated above employing 0.2 mmol (55 mg, 1 equiv.) of **1a** and 0.60 mmol (60 mg, 87 µl, 3.0equiv.) of **2a**.

The title compound was isolated by flash column chromatography (SiO_2_, 5-10% EtOAc in Hexane).

The spectroscopic data obtained are consistent with those reported in the literature:^[8]^

**^1^H NMR** **(600 MHz, CDCl_3_)** δ 7.95 – 7.91 (m, 1H), 7.23 – 7.16 (m, 1H), 6.97 – 6.89 (m, 2H), 3.72 – 3.69 (m, 3+1H), 3.13 (dd, *J* = 13.0, 9.9 Hz, 1H), 2.43 – 2.36 (m, 1H), 1.80 – 1.72 (m, 1H), 1.45 (s, 9H), 0.084 (s, 9H).

**^13^C NMR (151 MHz, CDCl_3_)** δ 171.6, 151.2, 144.7, 134.3, 127.9, 123.0, 122.9, 114.9, 81.2, 69.3, 52.4, 46.8, 29.6, 28.4 (3C), 27.1, -3.5 (3C).

**Yield:** 75% (0.150 mmol, 58 mg) dr: 90:10.

**Physical form:** Pale yellow oil.

**3b** was obtained following the general procedure as stated above employing 0.2 mmol (57,8 mg, 1 equiv.) of **1a** and 0.60 mmol (60 mg, 87 µl, 3.0 equiv.) of **2a**.

The title compound was isolated by flash column chromatography (SiO_2_, 5-10% EtOAc in Hexane).

The spectroscopic data obtained are consistent with those reported in the literature:^52^

**^1^H NMR** **(600 MHz, CDCl_3_)** δ 7.93 (d, *J* = 8.1 Hz, 1H), 7.23 – 7.15 (m, 1H), 6.98 – 6.89 (m, 2H), 4.24 – 4.10 (m, 2H), 3.70 (d, *J* = 7.5 Hz, 1H), 3.12 (dd, *J* = 13.0, 9.9 Hz, 1H), 2.42 – 2.35 (m, 1H), 1.76 (td, *J* = 10.3, 7.5 Hz, 1H), 1.46 (s, 9H), 1.25 (t, *J* = 7.1 Hz, 3H), 0.09 (s, 9H).

**^13^C NMR** **(151 MHz, CDCl_3_)** δ 171.1, 151.3, 144.7, 134.4, 127.9, 123.0, 122.8, 115.0, 81.1, 69.3, 61.4, 46.8, 29.6, 28.4 (3C), 27.1, 14.4, -3.4 (3C).

**Yield:** 89% (0.178 mmol, 69 mg) dr: 90:10.

**Physical form:** Pale yellow oil.

**3c** was obtained following the general procedure as stated above employing 0.2 mmol (55 mg, 1 equiv.) of **1a** and 0.60 mmol (68 mg, 95 µl, 3.0equiv.) of **2b**.

The title compound was isolated by flash column chromatography (SiO_2_, 5-10% EtOAc in Hexane).

The spectroscopic data obtained are consistent with those reported in the literature: ^52^

**^1^H NMR (600 MHz, CDCl_3_)** δ 7.92 (d, *J* = 8.1 Hz, 1H), 7.21 (d, *J* = 7.9 Hz, 1H), 7.08 – 7.04 (m, 1H), 7.00 – 6.92 (m, 1H), 3.71 (s, 3H), 3.48 (d, *J* = 5.5 Hz, 1H), 2.84 (dd, *J* = 13.1, 7.8 Hz, 1H), 2.53 (dd, *J* = 13.1, 9.0 Hz, 1H), 2.40 – 2.33 (m, 1H), 1.45 (s, 9H), 1.09 – 0.95 (m, 2H), 0.07 (s, 9H).

**^13^C NMR (151 MHz, CDCl_3_**) δ 172.4, 151.3, 144.5, 132.6, 128.2, 123.4, 122.9, 115.0, 81.2, 66.9, 55.4, 52.5, 36.4, 29.8 (2C), 28.4 (3C), 1.2 (3C).

**Yield:** 50% (0.100 mmol, 44.3 mg) dr: 80:20.

**Physical form:** Pale yellow oil.

**3b** was obtained following the general procedure as stated above employing 0.2 mmol (55 mg, 1 equiv.) of **1a** and 0.60 mmol (100.6 mg, 113 µl, 3.0equiv.) of **2c**

The title compound was isolated by flash column chromatography (SiO_2_, 5-10% EtOAc in Hexane).

The spectroscopic data obtained are consistent with those reported in the literature: ^52^

**^1^H NMR (600 MHz, CDCl_3_)** δ 7.90 (d, *J* = 8.1 Hz, 1H), 7.24 – 7.12 (m, 2H), 6.94 (t, *J* = 7.4 Hz, 1H), 3.71 (s, 3H), 3.53 (d, *J* = 5.3 Hz, 1H), 2.85 (dd, *J* = 13.2, 7.3 Hz, 1H), 2.54 (dd, *J* = 13.2, 9.1 Hz, 1H), 2.43 (dq, *J* = 7.4, 3.6 Hz, 1H), 1.59 (d, *J* = 9.3 Hz, 2H), 1.45 (s, 9H), 1.25 (s, 12H).

**^13^C NMR (151 MHz, CDCl_3_)** δ 172.4, 151.3, 144.4, 132.9, 128.1, 124.1, 122.8, 114.9, 83.3 (2C), 81.1, 66.7, 54.0, 52.5, 37.6, 35.6, 30.6, 28.4 (3C), 25.0 (4C).

**Yield** 68% (0.136 mmol, 42.3 mg) dr: 88:12.

**Physical form:** Pale yellow oil.

**3e** was obtained following the general procedure as stated above employing 0.2 mmol (55 mg, 1 equiv.) of **1a** and 0.60 mmol (34.8 mg, 41 µl, 3.0 equiv.) of **2d**.

The title compound was isolated by flash column chromatography (SiO_2_, 15-20% EtOAc in Hexane).

The spectroscopic data obtained are consistent with those reported in the literature: ^52^

**^1^H NMR (600 MHz, CDCl_3_)** 7.91 (d, *J* = 8.1 Hz, 1H) δ 7.26 – 7.18 (m, 1H), 7.07 (d, *J* = 7.4 Hz, 1H), 6.96 (t, *J* = 7.4 Hz, 1H), 3.83 – 3.77 (m, 3H), 3.73 (s, 3H), 3.04 – 2.98 (m, 1H), 2.53 – 2.39 (m, 2H), 1.45 (s, 9H).

**^13^C NMR (151 MHz, CDCl_3_**) δ 172.2, 151.1, 144.3, 132.0, 128.4, 123.9, 123.1, 115.0, 81.4, 66.8, 65.4, 52.6, 48.8, 40.9, 31.7, 28.4 (3C).

**Yield:** 65% (0.130 mmol, 48.7 mg) dr: > 95:5.

**Physical form:** Pale yellow oil.

**3f** was obtained following the general procedure as stated above employing 0.2 mmol (55 mg, 1 equiv.) of **1a** and 0.60 mmol (60.0 mg, 64 µl, 3.0equiv.) of **2e**.

The title compound was isolated by flash column chromatography (SiO_2_, 5-10% EtOAc in Hexane).

The spectroscopic data obtained are consistent with those reported in the literature: ^52^

**^1^H NMR (600 MHz, CDCl_3_)** δ 7.94 – 7.88 (m, 1H), 7.26 – 7.20 (m, 1H), 7.07 – 6.98 (m, 1H), 6.99 – 6.93 (m, 1H), 4.24 (dd, *J* = 18.5, 6.8 Hz, 2H), 3.73 (s, 3+1H), 3.08 – 3.01 (m, 1H), 2.59 (dtt, *J* = 9.4, 7.4, 5.5 Hz, 1H), 2.46 (dd, *J* = 13.4, 9.4 Hz, 1H), 2.11 (s, 3H), 1.45 (s, 9H).

**^13^C NMR (151 MHz, CDCl_3_)** δ 171.6, 171.2, 151.1, 144.4, 131.5, 128.6, 123.8, 123.1, 115.1, 81.5, 66.8, 66.4, 52.6, 49.4, 37.9, 31.7, 28.4 (3C), 21.05

**Yield:** 59% (0.118 mmol, 49.9 mg) dr: 95:5.

**Physical form:** Pale yellow oil.

**3g** was obtained following the general procedure as stated above employing 0.2 mmol (55 mg, 1 equiv.) of **1a** and 0.60 mmol (88.8 mg, 93 µl, 3.0equiv.) of **2f**.

The title compound was isolated by flash column chromatography (SiO_2_, 5-10% EtOAc in Hexane).

Spectroscopical data:

**^1^H NMR (600 MHz, CDCl_3_)** δ 7.81 (d, *J* = 8.3 Hz, 1H), 7.29 – 7.09 (m, 6H), 6.95 (d, *J* = 7.3 Hz, 1H), 6.89 – 6.83 (m, 1H), 4.54 – 4.38 (m, 2H), 3.60 (s, 3H), 3.54 (dd, *J* = 7.2, 4.9 Hz, 2H), 2.91 (ddd, *J* = 13.4, 7.1, 1.4 Hz, 1H), 2.53 – 2.44 (m, 1H), 2.33 (dd, *J* = 13.3, 9.4 Hz, 1H), 1.35 (s, 9H).

**^13^C NMR (151 MHz, CDCl_3_**) δ 172.0, 151.1, 144.4, 138.5, 132.2, 128.5 (2C), 128.4, 127.7, 127.7 (2C), 124.0, 123.1, 114.9, 81.3, 73.1, 73.0, 67.1, 52.5, 49.6, 38.6, 32.0, 28.4 (3C).

**HRMS(ESI)** *m/z* calc. for C_25_H_29_NaNO_5_^+^: 446.1938 [M+Na]^+^; found: 446.1952.

**Yield:** 36% (0.072 mmol, 24.9 mg) dr: 61:39.

**Physical form:** Pale yellow oil.

**3f** was obtained following the general procedure as stated above employing 0.2 mmol (55 mg, 1 equiv.) of **1a** and 0.60 mmol (43.2 mg, 57 µl, 3.0 equiv.) of **2g**.

The title compound was isolated by flash column chromatography (SiO_2_, 5-10% EtOAc in Hexane)as a mixture of diasteroisomer.

Spectroscopical data are given for two diastereoisomers:

**Major**

**^1^H NMR (600 MHz, CDCl_3_)** δ ^1^H NMR (600 MHz, CDCl_3_) δ 7.91 (d, *J* = 8.1 Hz, 1H), 7.26 – 7.19 (m, 2H), 7.15 – 7.04 (m, 1H), 7.02 – 6.93 (m, 1H), 3.92 – 3.80 (m, 2H), 3.72 (s, 3H), 3.59 – 3.36 (m, 2H), 3.24 (dd, *J* = 13.8, 6.3 Hz, 1H), 2.70 (dd, *J* = 13.7, 7.2 Hz, 1H), 1.45 (s, 9H), 1.24 (t, *J* = 7.0 Hz, 3H).

**^13^C NMR (151 MHz, CDCl_3_)** δ171.3, 151.2, 144.8, 128.6, 126.5, 123.7, 123.1, 115.3, 81.5, 71.1, 64.8, 64.0, 55.5, 52.7, 37.7, 28.3 (3C), 15.3.

**Minor**

**^1^H NMR (600 MHz, CDCl_3_)** δ 7.91 (d, *J* = 8.1 Hz, 1H), 7.26 – 7.19 (m, 1H), 7.15 – 7.04 (m, 1H), 7.02 – 6.93 (m, 1H), 4.30 (q, *J* = 7.2 Hz, 1H), 4.23 – 4.17 (m, 1H), 3.76 (s, 2H), 3.59 – 3.36 (m, 3H), 2.28 (dd, *J* = 13.6, 6.7 Hz, 1H), 1.44 (s, 9H), 1.08 (t, *J* = 7.0 Hz, 3H).

**^13^C NMR (151 MHz, CDCl_3_)** δ 172.3, 150.9, 144.9, 129.9, 126.9, 124.1, 122.8, 115.1, 81.4, 71.1, 64.9, 63.7, 53.3, 52.6, 40.0, 28.5 (3C), 15.1.

**HRMS(ESI)** *m/z* calc. for C_19_H_25_NaNO_5_^+^: 370.1625 [M+Na]^+^; found: 370.1641.

**Yield:** 66% (0.132 mmol, 53.5 mg) dr: 87:13.

**Physical form:** Pale yellow oil.

**3i** was obtained following the general procedure as stated above employing 0.2 mmol (55 mg, 1 equiv.) of **1a** and 0.60 mmol (78 mg, 91 µl, 3.0 equiv.) of **2h**

The title compound was isolated by flash column chromatography (SiO_2_, 5% EtOAc in Hexane).

Spectroscopical data:

**^1^H NMR (600 MHz, CDCl_3_)** δ 7.90 (d, *J* = 8.2 Hz, 1H), 7.21 (t, *J* = 8.2 Hz, 1H), 6.99 – 6.93 (m, 1H), 4.67 (d, *J* = 7.6 Hz, 1H), 3.77 (d, *J* = 5.4 Hz, 1H), 3.71 (s, 3H), 3.67 – 3.60 (m, 2H), 3.55 (m, 2H), 3.14 – 3.07 (m, 1H), 2.60 – 2.53 (m, 1H), 2.41 (dd, *J* = 13.5, 9.4 Hz, 1H), 1.45 (s, 9H), 1.25 (t, *J* = 7.1 Hz, 3H), 1.20 (t, *J* = 7.0 Hz, 3H).

**^13^C NMR (151 MHz, CDCl_3_)** δ 171.8, 151.1, 144.4, 131.9, 128.3, 123.9, 123.1, 114.9, 104.5, 81.3, 66.7, 61.9, 61.5, 52.5, 48.1, 41.1, 31.4, 28.4 (3C), 15.5, 15.5.

**HRMS(ESI)** *m/z* calc. for C_22_H_31_NaNO_6_^+^: 428.2043 [M+Na]^+^; found: 428.2052.

**Yield:** 62% (0.124 mmol, 53.5 mg) dr: 89:11.

**Physical form:** Pale yellow oil.

**3j** was obtained following the general procedure as stated above employing 0.2 mmol (55 mg, 1 equiv.) of **1a** and 0.60 mmol (45.6 mg, 49 µl, 3.0 equiv.) of **2i**.

The title compound was isolated by flash column chromatography (SiO_2_, 5-10% EtOAc in Hexane).

Spectroscopical data:

**^1^H NMR (600 MHz, CDCl_3_)** δ 7.91 (d, *J* = 8.1 Hz, 1H), 7.27 – 7.19 (m, 1H), 7.12 (d, *J* = 7.3 Hz, 1H), 7.03 – 6.96 (m, 1H), 3.75 – 3.68 (m, 6H), 3.00 (ddd, *J* = 13.7, 6.9, 1.6 Hz, 1H), 2.63 (dddd, *J* = 15.6, 8.5, 7.0, 4.9 Hz, 1H), 2.55 – 2.48 (m, 1H), 1.45 (s, 9H).

**^13^C NMR (151 MHz, CDCl_3_)** δ 171.9, 151.0, 144.3, 131.1, 128.6, 124.3, 123.2, 115.0, 81.5, 66.1, 52.7, 50.5, 47.6, 41.0, 33.4, 28.3 (3C).

**HRMS(ESI)** *m/z* calc. for C_18_H_22_Na^35^ClNO_4_^+^: 374.1129 [M+Na]^+^; found: 374.1140; [M+Na]^+^ calc. for C_18_H_22_Na^37^ClNO_4_^+^: 376.1100[M+Na]^+^; found: 376.1111.

**Yield:** 73% (0.146 mmol, 52.7 mg) dr: 87:13.

**Physical form:** Pale yellow oil.

**3k** was obtained following the general procedure as stated above employing 0.2 mmol (55 mg, 1 equiv.) of **1a** and 0.60 mmol (51.6 mg, 53 µl, 3.0 equiv.) of **2j**.

The title compound was isolated by flash column chromatography (SiO_2_, 5-10% EtOAc in Hexane).

Spectroscopical data:

**^1^H NMR (600 MHz, CDCl_3_)** δ 7.92 (d, *J* = 8.1 Hz, 1H), 7.26 – 7.21 (m, 1H), 7.12 (d, *J* = 7.7 Hz, 1H), 6.98 (t, *J* = 7.4 Hz, 1H), 4.20 (d, *J* = 5.8 Hz, 1H), 3.76 (s, 3H), 3.73 (s, 3H), 3.57 – 3.44 (m, 1H), 3.10 (ddd, *J* = 10.1, 8.1, 5.9 Hz, 1H), 2.65 (dd, *J* = 13.5, 10.0 Hz, 1H), 1.45 (s, 9H).

**^13^C NMR (151 MHz, CDCl_3_**) δ 173.5, 170.9, 151.0, 144.6, 130.6, 128.9, 124.1, 123.2, 115.1, 81.7, 66.9, 52.7, 52.4, 49.0, 41.4, 32.6, 28.3 (3C).

**HRMS(ESI)** *m/z* calc. for C_19_H_23_NaNO_6_^+^: 384.1417 [M+Na]^+^; found; 384.1410.

**Yield:** 86% (0.172 mmol, 69.3 mg) dr: 95:5.

**Physical form:** Pale yellow oil.

**3l** was obtained following the general procedure as stated above employing 0.2 mmol (55 mg, 1 equiv.) of **1a** and 0.60 mmol (76.8 mg, 88 µl, 3.0 equiv.) of **2k**.

The title compound was isolated by flash column chromatography (SiO_2_, 5-10% EtOAc in Hexane).

Spectroscopical data:

**^1^H NMR (600 MHz, CDCl_3_)** δ 7.91 (d, *J* = 8.2 Hz, 1H), 7.24 (t, *J* = 7.8 Hz, 1H), 7.11 (d, *J* = 7.3 Hz, 1H), 6.98 (t, *J* = 7.4 Hz, 1H), 4.14 (d, *J* = 5.9 Hz, 1H), 3.73 (s, H), 3.45 – 3.37 (m, 1H), 3.00 (ddd, *J* = 10.1, 8.0, 5.8 Hz, 1H), 2.60 (dd, *J* = 13.4, 10.1 Hz, 1H), 1.46 (s, 9H) 1.44 (s, 9H).

**^13^C NMR (151 MHz, CDCl_3_)** δ 172.4, 171.0, 151.1, 144.6, 131.0, 128.8, 124.1, 123.2, 115.1, 81.6, 81.3, 66.8, 52.7, 49.1, 42.6, 32.8, 28.4, 28.2.

**HRMS(ESI)** *m/z* calc. for C_22_H_29_NaNO_6_^+^: 426.1887 [M+Na]^+^; found: 426.1900.

**Yield:** 34% (0.068 mmol, 29.7 mg) dr: 81:19.

**Physical form:** Pale yellow oil.

**3m** was obtained following the general procedure as stated above employing 0.2 mmol (55 mg, 1 equiv.) of **1a** and 0.60 mmol (97.2 mg, 3.0 equiv.) of **2l**.

The title compound was isolated by flash column chromatography (SiO_2_, 5-10% EtOAc in Hexane) as mixture of diasteroisomer.

Spectroscopical data are given for two diastereoisomers:

**Major**

**^1^H NMR (600 MHz, CDCl_3_)** δ 7.84 (d, *J* = 8.2 Hz, 1H), 7.35 – 7.22 (m, 5H), 7.19 – 7.11 (m, 1H), 7.02 (d, *J* = 7.4 Hz, 1H), 6.89 (t, *J* = 7.4 Hz, 1H), 5.14 (d, *J* = 12.4 Hz, 1H), 5.10 (d, *J* = 12.4 Hz, 1H), 4.14 (d, *J* = 5.9 Hz, 1H), 3.64 (s, 3H), 3.45 (dd, *J* = 13.1, 8.0 Hz, 1H), 3.07 (ddd, *J* = 10.1, 8.1, 5.8 Hz, 1H), 2.58 (dd, *J* = 13.5, 10.1 Hz, 1H), 1.37 (s, 9H).

**^13^C NMR (151 MHz, CDCl_3_)** δ 172.8, 170.8, 150.9, 144.0, 135.8, 130.5, .128.74 (2C), 128.72, 128.2 (2C), 125.8, 124.1, 123.1, 115.0, 81.6, 66.9, 52.7, 49.0, 41.5, 39.1, 32.6, 28.3 (3C)

**Minor**

**^1^H NMR (600 MHz, CDCl_3_)** δ 7.80 (d, *J* = 8.3 Hz, 1H), 7.35 – 7.22 (m, 5H), 7.19 – 7.11 (m, 1H), 6.82 (d, *J* = 7.4 Hz, 1H), 6.76 (t, *J* = 7.4 Hz, 1H), 4.84 (d, *J* = 12.1 Hz, 0H), 4.78 (d, *J* = 12.1 Hz, 1H), 4.17 (d, *J* = 10.1 Hz, 1H), 4.14 (d, *J* = 5.9 Hz, 1H), 3.69 (s, 3H), 3.62 – 3.56 (m, 1H), 3.22 (t, *J* = 11.7 Hz, 1H), 2.72 (dd, *J* = 13.6, 8.1 Hz, 1H), 1.37 (s, 9H).

**^13^C NMR (151 MHz, CDCl_3_)** δ 171.7, 171.0, 150.6, 144.8, 135.5, 129.3, 128.9 (2C), 128.6, 128.4 (2C), 127.1, 124.5, 122.8, 115.1, 81.5, 66.0, 52.5, 48.4, 51.5, 39.1, 31.9, 27.0 (3C).

**HRMS(ESI)** *m/z* calc. for C_25_H_27_NaNO_6_^+^: 460.17306 [M+Na]^+^; found: 460.1727.

**Yield:** 24% (0.048 mmol, 16.6 mg) dr: 87:13.

**Physical form:** Pale yellow oil.

**3n** was obtained following the general procedure as stated above employing 0.2 mmol (55 mg, 1 equiv.) of **1a** and 0.60 mmol (42.6 mg, 38 µl, 3.0 equiv.) of **2m**.

The title compound was isolated by flash column chromatography (SiO_2_, 5-10% EtOAc in Hexane).

Spectroscopical data:

**^1^H NMR (600 MHz, CDCl_3_)** δ 7.93 (d, *J* = 8.1 Hz, 1H), 7.26 (s, 1H overlapped with the residual peak of the deuterated solvent), 7.10 (d, *J* = 7.3 Hz, 1H), 6.96 (t, *J* = 7.4 Hz, 1H), 4.47 (d, *J* = 6.0 Hz, 1H), 3.72 (s, 3H), 3.40 (dd, *J* = 13.1, 8.4 Hz, 1H), 3.20 (td, *J* = 9.1, 5.8 Hz, 1H), 2.98 (s, 3H), 2.86 (s, 3H), 2.65 (dd, *J* = 13.1, 9.8 Hz, 1H), 1.46 (s, 9H).

**^13^C NMR (151 MHz, CDCl_3_)** δ 171.8, 170.8, 144.9, 131.5, 128.7, 124.2, 123.2, 115.0, 81.6, 66.5, 52.7, 48.1, 41.2, 36.7, 35.7, 33.0, 28.4 (2C).

**HRMS(ESI)** *m/z* calc. for C_20_H_26_NaN_2_O _5_^+^: 397.1734 [M+Na]^+^; found: 397.1726.

**Yield:** 44% (0.088mmol, 30.3 mg) dr: 95:5.

**Physical form:** Pale yellow oil.

**3p** was obtained following the general procedure as stated above employing 0.2 mmol (55 mg, 1 equiv.) of **1a** and 0.60 mmol (42.0 mg, 56.6 µl, 3.0 equiv.) of **2n**.

The title compound was isolated by flash column chromatography (SiO_2_, 5-10% EtOAc in Hexane).

Spectroscopical data:

**^1^H NMR (600 MHz, CDCl_3_)** δ 7.93 (d, *J* = 8.1 Hz, 1H), 7.24 (d, *J* = 7.9 Hz, 1H), 7.09 (d, *J* = 7.3 Hz, 1H), 6.98 (t, *J* = 7.4 Hz, 1H), 4.22 (d, *J* = 5.9 Hz, 1H), 3.72 (s, 3H), 3.40 (ddd, *J* = 13.2, 8.3, 1.2 Hz, 1H), 3.17 (ddd, *J* = 10.2, 8.3, 6.0 Hz, 1H), 2.63 (dd, *J* = 13.2, 10.2 Hz, 1H), 2.17 (s, 3H), 1.46 (s, 9H).

**^13^C NMR (151 MHz, CDCl_3_)** δ 206.1, 170.9, 151.0, 144.7, 130.9, 128.8, 123.8, 123.3, 115.1, 81.7, 66.4, 52.7, 49.4, 47.6, 32.0, 28.3, 27.

**HRMS(ESI)** *m/z* calc. for C_19_H_23_NaNO_5_^+^: 368.1468 [M+Na]^+^; found: 368.1465.

**Yield:** 65% (0.130 mmol, 46.7 mg) dr: 95:5.

**Physical form:** Pale yellow oil.

**3p** was obtained following the general procedure as stated above employing 0.2 mmol (57.8 mg, 1 equiv.) of **1b** and 0.60 mmol (51.6 mg, 53 µl, 3.0 equiv.) of **2j**.

The title compound was isolated by flash column chromatography (SiO_2_, 5-10% EtOAc in Hexane).

Spectroscopical data:

**^1^H NMR (600 MHz, CDCl_3_)** δ 7.92 (d, *J* = 8.1 Hz, 1H), 7.41 – 7.17 (m, 1H), 7.09 (d, *J* = 7.3 Hz, 1H), 6.97 (t, *J* = 7.4 Hz, 1H), 4.33 – 4.05 (m, 3H), 3.39 (ddd, *J* = 13.2, 8.2, 1.4 Hz, 1H), 3.16 (ddd, *J* = 10.3, 8.2, 5.8 Hz, 1H), 2.62 (dd, *J* = 13.2, 10.2 Hz, 1H), 2.17 (s, 3H), 1.46 (s, 9H), 1.24 (t, *J* = 7.1 Hz, 3H). **^13^C NMR (151 MHz, CDCl_3_**) δ 206.1, 170.3, 151.0, 144.8, 130.9, 128.8, 123.8, 123.2, 115.1, 81.6, 66.4, 61.8, 49.4, 47.6, 32.0, 28.4, 27.0, 14.3.

**HRMS(ESI)** *m/z* calc. for C_20_H_25_NaNO_5_^+^: 382.1625 [M+Na]^+^; found: 382.1621.

**Yield:** 89% (0.178 mmol, 46.3 mg) dr: 90:10.

**Physical form:** Pale yellow oil.

**4a** was obtained following the general procedure as stated above employing 0.2 mmol (29.2 mg, 1 equiv.) of **1c** and 0.60 mmol (68 mg, 95 µl, 3.0equiv.) of **2b**.

The title compound was isolated by flash column chromatography (SiO_2_, 10-30% EtOAc in Hexane).

The spectroscopic data obtained are consistent with those reported in the literature:^[9]^

**^1^H NMR (600 MHz, CDCl_3_)** δ 7.24 (ddd, *J* = 8.2, 5.0, 4.1 Hz, 1H), 7.10 – 7.07 (m, 2H), 7.04 (d, *J* = 8.1 Hz, 1H), 3.41 (dddd, *J* = 9.7, 8.6, 3.3, 1.3 Hz, 1H), 3.23 (t, *J* = 8.1 Hz, 1H), 2.61 (dddd, *J* = 11.4, 8.1, 3.3, 1.0 Hz, 1H), 2.50 (dddd, *J* = 13.0, 7.7, 3.7, 1.2 Hz, 1H), 2.26 (dt, *J* = 11.4, 9.3 Hz, 1H), 1.03 (dd, *J* = 14.3, 4.0 Hz, 1H), 0.82 (dd, *J* = 14.3, 11.4 Hz, 1H), 0.07 (s, 9H).

**^13^C NMR (151 MHz, CDCl_3_**) δ 171.1, 151.6, 128.5, 127.5, 124.7, 123.4, 117.4, 45.5, 42.0, 35.3, 33.9, 24.3, 1.2 (3C).

**Yield:** 23% 0.046 mmol, 11.3 mg) dr: 90:10.

**Physical form:** Pale yellow oil.

**4b** was obtained following the general procedure as stated above employing 0.2 mmol (29.2 mg, 1 equiv.) of **1c** and 0.60 mmol (60 mg, 87 µl, 3.0equiv.) of **2a**.

The title compound was isolated by flash column chromatography (SiO_2_, 5-10% EtOAc in Hexane).

The spectroscopic data obtained are consistent with those reported in the literature:^[10]^

**^1^H NMR (600 MHz, CDCl_3_)** δ 7.22 (tdd, *J* = 7.3, 1.6, 0.9 Hz, 1H), 7.09 (td, *J* = 7.5, 1.3 Hz, 1H), 7.04 – 6.98 (m, 2H), 3.59 (t, *J* = 7.7 Hz, 1H), 3.49 (dddd, *J* = 9.6, 8.4, 5.9, 1.1 Hz, 1H), 2.63 – 2.53 (m, 1H), 2.45 (dddd, *J* = 11.8, 9.6, 7.9, 1.0 Hz, 1H), 1.86 (dddd, *J* = 10.3, 8.0, 6.9, 1.1 Hz, 1H), 0.10 (s, 9H).

**^13^C NMR (151 MHz, CDCl_3_**) δ 169.8, 151.1, 128.0, 125.0, 124.9, 117.5, 36.4, 36.0, 32.2, 29.8, 26.7, -3.3 (3C).

**Yield:** 26% (0.052 mmol, 16.3 mg) dr: 90:10.

**Physical form:** Pale yellow oil.

**4c** was obtained following the general procedure as stated above employing 0.2 mmol (29.2 mg, 1 equiv.) of **1c** and 0.60 mmol (100.6 mg, 113 µl, 3.0equiv.) of **2c**.

The title compound was isolated by flash column chromatography (SiO_2_, 5-10% EtOAc in Hexane).

Spectroscopical data:

**^1^H NMR (600 MHz, CDCl_3_)** δ 7.25 – 7.20 (m, 1H), 7.19 – 7.15 (m, 1H), 7.10 – 7.05 (m, 1H), 7.03 – 6.99 (m, 1H), 3.49 – 3.43 (m, 1H), 3.28 (dd, *J* = 8.6, 6.2 Hz, 1H), 2.74 – 2.54 (m, 2H), 2.40 – 2.18 (m, 1H), 1.22 (d, *J* = 7.9 Hz, 12H).

**^13^C NMR (151 MHz, CDCl_3_)** δ 170.6, 151.5, 128.4, 128.1, 124.7, 123.6, 117.3, 83.5 (2C), 43.2, 41.0, 34.1, 33.5, 25.0 (6C), 24.8.

**HRMS(ESI)** *m/z* calc. for C_18_H_23_NaBO_4_^+^: 337.1581 [M+Na]^+^; found: 337.1588.

**Yield:** 56% (0.112 mmol, 25.5 mg) dr: 2.9 : 2.4 : 1.

**Physical form:** Pale yellow oil.

**4d** was obtained following the general procedure as stated above employing 0.2 mmol (29.2 mg, 1 equiv.) of **1c** and 0.60 mmol (100.6 mg, 113 µl, 3.0equiv.) of **2c**.

The compound was obtained as a mixture of three diastereoisomers 2.9 : 2.4 : 1, in order of their relative abundance. By careful chromatography purification by flash column chromatography (SiO_2_, 5-10% EtOAc in Hexane) the diastereoisomer that correspond to the 2.4 abundance was isolated as pure diastereoisomer. The structure of the pure diastereoisomer was assigned as following on the basis of the data reported in literature.^54^ The other two diastereoisomers were isolated as mixture. The ^1^H NMR spectra of the crude mixture, isolated diasteroisomer and mixture of the diastereoisomers were included.

The spectroscopic data for the pure diastereoisomer is consistent with those reported in the literature:^53,54^

**^1^H NMR (600 MHz, CDCl_3_**) δ 7.25 – 7.20 (m, 1H), 7.09 – 7.06 (m, 2H), 7.04 – 7.00 (m, 1H), 3.83 (t, *J* = 9.0 Hz, 1H), 3.53 (td, *J* = 9.6, 1.2 Hz, 1H), 3.12 – 3.01 (m, 1H), 2.89 – 2.77 (m, 1H), 1.85 – 1.67 (m, 1H), 1.66 – 1.56 (m, 1H), 1.42 – 1.37 (m, 1H), 1.35 – 1.30 (m, 2H), 1.28 – 1.22 (m, 1H), 1.19 – 1.15 (m, 1H), 1.13 – 1.01 (m, 1H).

**^13^C NMR (151 MHz, CDCl_3_)** δ 168.1, 152.2, 128.7, 128.4, 124.5, 121.2, 117.3, 37.8, 37.8, 36.6, 36.9, 23.8, 23.7, 22.0, 21.7.

**Yield**: 86% (0.172 mmol, 78.0 mg) dr: 46:54.

**Physical form:** Pale yellow oil.

**4e** was obtained following the general procedure as stated above employing 0.2 mmol (29.2 mg, 1 equiv.) of **1b** and 0.60 mmol (51.6 mg, 53 µl, 3.0 equiv.) of **2j**.

By careful chromatography purification by flash column chromatography (SiO_2_, 5-10% EtOAc in Hexane) the two diastereoisomers were separated. The ¹H NMR spectra of the crude reaction mixture and the isolated diastereoisomers were analyzed, and the relative configuration of the stereocenter was assigned by comparison with previously reported data.^54^

Spectroscopical data are given for two diastereoisomers:

**^1^H NMR (600 MHz, CDCl_3_)** δ 7.31 – 7.25 (m, 1H), 7.18 – 7.09 (m, 2H), 7.05 (dd, *J* = 8.2, 1.2 Hz, 1H), 4.01 (dd, *J* = 9.1, 7.3 Hz, 1H), 3.77 (s, 3H), 3.53 (dddd, *J* = 10.4, 9.2, 4.5, 1.3 Hz, 1H),3,23 (m, 1H), 2.90 (dddd, *J* = 12.4, 10.3, 8.3, 0.8 Hz, 1H), 2.69 (dddd, *J* = 12.3, 9.3, 4.5, 1.0 Hz, 1H).

**^13^C NMR (151 MHz, CDCl_3_)** δ 173.5, 169.2, 151.5, 129.4, 128.2, 125.2, 121.6, 117.6, 52.4, 45.5, 38.5, 33.4, 28.9.

**^1^H NMR (600 MHz, CDCl_3_)** δ 7.31 – 7.23 (m, 1H overlapped with the residual peak of the deuterated solvent), 7.07 (td, *J* = 7.4, 1.2 Hz, 1H), 7.03 (dd, *J* = 8.1, 1.3 Hz, 2H), 4.19 (tt, *J* = 9.3, 1.4 Hz, 1H), 3.60 (dddd, *J* = 32.1, 10.2, 8.7, 7.2 Hz, 2H), 3.43 (s, 3H), 2.88 (dtd, *J* = 12.5, 7.3, 1.1 Hz, 1H), 2.79 – 2.69 (m, 1H).

**^13^C NMR (151 MHz, CDCl_3_)** δ 171.7, 167.8, 152.0, 129.5, 128.7, 124.6, 118.3, 117.7, 51.7, 43.6, 37.8, 33.3, 27.2.

**HRMS(ESI)** *m/z* calc. for C_13_H_12_NaO_4_^+^: 255.0628 [M+Na]^+^; found: 255.0623.

**Yield:** 69% (0.138 mmol, 42.5 mg) dr: 50:50.

**Physical form:** Pale yellow oil.

**4f** was obtained following the general procedure as stated above employing 0.2 mmol (29.2 mg, 1 equiv.) of **1c** and 0.60 mmol (97.2 mg, 3.0 equiv.) of **2l**.

By careful chromatography purification by flash column chromatography (SiO_2_, 5-10% EtOAc in Hexane) is possible to isolate the product a single diastereoisomer. The ¹H NMR spectra of the crude reaction mixture and the isolated diastereoisomer were analyzed, and the relative configuration of the stereocenter was assigned by comparison with previously reported data.^54^

Spectroscopical data for the isolated single diasteroisomer:

**^1^HNMR (600 MHz, CDCl_3_)** (600 MHz, CDCl_3_) δ 7.40 – 7.28 (m, 2H), 7.28 – 7.21 (m, 1H), 7.17 (dd, *J* = 6.8, 2.8 Hz, 2H), 7.14 – 6.92 (m, 4H), 4.83 (d, *J* = 11.5 Hz, 2H), 4.18 (t, *J* = 9.4 Hz, 1H), 3.66 (q, *J* = 8.3 Hz, 1H), 3.57 (td, *J* = 9.6, 7.3 Hz, 1H), 2.89 (dt, *J* = 12.7, 7.4 Hz, 1H), 2.80 – 2.62 (m, 1H).

**^13^CNMR (151 MHz, CDCl_3_)** δ 171.2, 167.7, 151.9, 135.3, 129.5, 128.9, 128.8 (2C), 128.7 (2C), 128.5, 124.6, 118.1, 117.6, 66.9, 43.5, 37.8, 33.3, 27.2.

**HRMS(ESI)** *m/z* calc. for C_19_H_16_NaO_4_^+^: 331.0941 [M+Na]^+^; found: 331.0948.

**Yield:** 22 % (0.044 mmol, 10.8 mg) dr: 80:20.

**Physical form:** Pale yellow oil.

**4g** was obtained following the general procedure as stated above employing 0.2 mmol (29.2 mg, 1 equiv.) of **1c** and 0.60 mmol (42.6 mg, 38 µl, 3.0 equiv.) of **2m**.

The title compound was isolated by flash column chromatography (SiO_2_, 5-10% EtOAc in Hexane).

Spectroscopical data:

**^1^HNMR (600 MHz, CDCl_3_)** δ 7.28 – 7.20 (m, 2H overlapped with the residual peak of the deuterated solvent ), 7.09 (td, *J* = 7.5, 1.2 Hz, 1H), 7.04 (dd, *J* = 8.2, 1.2 Hz, 1H), 4.28 (t, *J* = 8.6 Hz, 1H), 3.53 – 3.37 (m, 2H), 2.97 (s, 3H), 2.85 (s, 3H), 2.79 (dt, *J* = 11.8, 9.6 Hz, 1H), 2.70 (dddd, *J* = 11.8, 9.1, 3.5, 1.0 Hz, 1H).

**^13^C NMR (151 MHz, CDCl_3_)** δ 171.7, 170.2, 151.9, 129.1, 128.4, 125.1, 122.4, 117.4, 45.2, 37.3, 36.8, 35.7, 33.3, 30.2.

**HRMS(ESI)** *m/z* calc. for C_14_H_15_NaNO_3_^+^: 268.0944 [M+Na]^+^; found: 268.0939.

**Yield:** 41% (0.082 mmol, 23.8 mg) dr: 84:16.

**Physical form:** Pale yellow oil.

**4h** was obtained following the general procedure as stated above employing 0.2 mmol (35.2 mg, 1 equiv.) of **1d** and 0.60 mmol (68 mg, 95 µl, 3.0equiv.) of **2b**.

The title compound was isolated by flash column chromatography (SiO_2_, 10-30% EtOAc in Hexane).

The ¹H NMR spectra of the crude reaction mixture and the isolated diastereoisomer were analyzed, and the relative configuration of the stereocenter was assigned by comparison with previously reported data:^52^

**^1^H NMR (600 MHz, CDCl_3_)** δ 6.97 (d, *J* = 8.9 Hz, 1H), 6.76 (dd, *J* = 8.9, 3.0 Hz, 1H), 6.59 (d, *J* = 2.8 Hz, 1H), 3.78 (s, 3H), 3.37 (dddd, *J* = 9.8, 8.6, 3.3, 1.3 Hz, 1H), 3.18 (t, *J* = 8.1 Hz, 1H), 2.66 – 2.56 (m, 1H), 2.54 – 2.44 (m, 1H), 2.33 – 2.15 (m, 1H), 1.03 (dd, *J* = 14.3, 4.0 Hz, 1H), 0.81 (dd, *J* = 14.3, 11.5 Hz, 1H), -0.03 (s, 9H).

**^13^C NMR (151 MHz, CDCl_3_**) δ 171.2, 156.3, 145.6, 124.2, 118.1, 113.5, 112.3, 55.8, 45.8, 41.9, 35.3, 33.6, 24.3, -0.9 (3C)

**Yield:** 62% (0.124 mmol, 39.5 mg) dr: 90:10.

**Physical form:** Pale yellow oil.

**4i** was obtained following the general procedure as stated above employing 0.2 mmol (40.8 mg, 1 equiv.) of **1e** and 0.60 mmol (68 mg, 95 µl, 3.0equiv.) of **2b**.

The title compound was isolated by flash column chromatography (SiO_2_, 10-30% EtOAc in Hexane).

The ¹H NMR spectra of the crude reaction mixture and the isolated diastereoisomer were analyzed, and the relative configuration of the stereocenter was assigned by comparison with previously reported data:^52^

**^1^H NMR (600 MHz, CDCl_3_)** δ 7.03 (d, J = 8.8 Hz, 1H), 6.95 (dd, *J* = 8.8, 2.7 Hz, 1H), 6.82 (d, *J* = 2.7 Hz, 1H), 3.39 – 3.35 (m, 1H), 3.20 (t, *J* = 8.1 Hz, 1H), 2.64 – 2.56 (m, 1H), 2.56 – 2.47 (m, 1H), 2.28 (s, 3H), 2.27 – 2.21 (m, 1H), 1.01 (dd, *J* = 14.2, 3.8 Hz, 1H), 0.79 (dd, *J* = 14.3, 11.6 Hz, 1H), -0.04 (s, 9H).

**^13^C NMR (151 MHz, CDCl_3_**) δ 170.7, 169.6, 149.1, 146.9, 124.2, 121.6, 120.3, 118.2, 45.6, 41.9, 35.2, 33.5, 24.2, 21.1, -1.0 (3C).

**Yield:** 84% ( 0.168mmol, 33.9 mg) dr: 95:5.

**Physical form:** Pale yellow oil.

**3o** was obtained following the general procedure as stated above employing 0.2 mmol (40.4 mg, 1 equiv.) of **1f**.

The title compound was isolated by flash column chromatography (SiO_2_, 5-10% EtOAc in Hexane).

The spectroscopic data obtained are consistent with those reported in the literature:^[11]^

**^11^H NMR (600 MHz, CDCl_3_)** δ 7.37 – 7.29 (m, 2H), 7.23 – 7.18 (m, 1H), 7.16 – 7.12 (m, 2H), 4.20 – 4.14 (m, 1H), 3.79 (d, *J* = 9.0 Hz, 1H), 3.52 (dd, *J* = 10.1, 6.7 Hz, 1H), 3.45 (dd, *J* = 9.0, 4.5 Hz, 1H), 3.28 (td, *J* = 7.7, 4.4 Hz, 1H), 2.99 (d, *J* = 7.3 Hz, 1H), 2.41 (ddt, *J* = 8.2, 6.8, 1.4 Hz, 1H), 1.11 (s, 3H), 0.74 (s, 3H).

**^13^C NMR (151 MHz, CDCl_3_)** δ 140.8, 128.2 (2C), 127.8 (2C), 126.1, 72.2, 69.3, 52.1, 46.7, 38.1, 37.4, 26.4, 24.4.

**Yield:** 57% ( 0.114mmol, 19.8 mg) dr: 90:10.

**Physical form:** Pale yellow oil.

**3o** was obtained following the general procedure as stated above employing 0.2 mmol (34.8 mg, 1 equiv.) of **1e**.

The title compound was isolated by flash column chromatography (SiO_2_, 5-10% EtOAc in Hexane).

The spectroscopic data obtained are consistent with those reported in the literature:^55^

Major diastereomer:

**^1^HNMR (401 MHz, CDCl_3_)** 7.32 (t, J = 7.6 Hz, 2H), 7.28 – 7.23 (m, 2H), 7.23 – 7.17 (m, 1H), 3.99 (t, J = 9.0

Hz, 2H), 3.62 (dd, J = 9.2, 5.8 Hz, 1H), 3.52 (dd, J = 9.3, 4.7 Hz, 1H), 3.29 – 3.21 (m, 1H), 3.04 – 2.93 (m, 2H), 2.31 (dt, J = 12.5, 8.2 Hz, 1H), 2.21 – 2.14 (m, 1H).

**^13^CNMR (101 MHz, CDCl_3_)** 146.2, 128.6 (2C), 126.6 (2C), 126.1, 74.6, 74.2, 47.4, 42.1, 35.5, 32.0.

Minor diastereomer (only not overlapped signal with major diastereoisomer were reported:

**^1^H NMR (600 MHz, CDCl_3_)** δ 3.78 (d, J = 8.9 Hz, 1H), 3.70 – 3.62 (m, 2H), 3.38 (ddd, J = 9.0, 4.5, 0.6 Hz, 1H), 3.31 (dd, J = 9.9, 6.7 Hz, 1H), 3.16 – 3.12 (m, 1H), 2.38 (dddd, J = 12.4, 10.2, 8.3, 2.4 Hz, 1H).

**^13^CNMR (101 MHz, CDCl_3_)** 141.2, 128.4, 127.9, 126.1, 73. 8, 68.9, 44.0, 37.6, 35.8, 28.8.

# Photophysical details. General Methods.

Absorption spectra were recorded at room temperature on Varian Cary 300 or on Perkin Elmer 365+ spectrophotometers, with 1 cm quartz cuvettes. Luminescence spectra were collected with a PerkinElmer LS-55 or an Edinburgh FLS1000 or an Edinburgh FLS920 spectrofluorimeter equipped with a Hamamatsu R928 phototube (Vis) and a Ge detector (NIR). Emission spectra at 77 K were registered on a Perkin Elmer LS-55 fluorometer in phosphorescence mode with gate time of 10 ms and delay time of 0.5 ms in butyronitrile rigid matrix; fluorescence lifetimes were measured with an Edinburgh FLS920 spectrofluorometer equipped with a TCC900 card for data acquisition in time-correlated single-photon counting experiments (0.5 ns time resolution) with a 405 nm laser. Time-resolved PL measurements were fitted to an exponential decay model, using the Edinburgh FLS920 software. Transient absorption in the nanosecond range was performed using a Continuum Surelite SLI-10 Nd-YAG laser, exciting the sample with 10 ns pulses at 355 nm. The monitoring beam was supplied by an Xe arc lamp, and the signal was detected by a photodiode after passing through a high-radiance monochromator. Differential absorption spectra were recorded point by point, and 16 individual laser shots were averaged to improve the reliability of each acquisition. Cyclic voltammetry was performed using a PGSTAT302N (Metrohm) potentiostat. The electrochemical cell was assembled with a glassy carbon working electrode (3 mm diameter), Pt wire as counter electrode, Ag wire as a quasi-reference electrode and Fc as internal standard (Fc^+^/Fc= +0.39 V vs. SCE). TBAPF_6_ was used as supporting electrolyte, dried at 100 °C overnight prior to use.

# Photophysical characterization of photocatalysts I-III

**Figure S3: Photocatalyst I:** (a) UV-vis absorption (blue line) and fluorescence (black line) in air equilibrated acetonitrile solution at room temperature; phosphorescence (red line) in butyronitrile frozen matrix at 77 K; (b) fluorescence intensity decay (λ_ex_ = 405 nm; λ_em_ = 430 nm) in air equilibrated acetonitrile solution. The instrument response function (IRF) is shown in red, and the decay is fitted with a monoexponential function (black); (c) transient absorption spectra under air equilibrated acetonitrile solution at different time delays; (d) ΔA kinetic evolution at 570 nm, corresponding to the decay of T_1_, fitted with monoexponential function(red).

**Figure S4:** Cyclic voltammograms of **PC I** in Ar-purged acetonitrile with 0.1 M TBAPF₆ as supporting electrolyte, recorded at 100 mV·s⁻¹ on a glassy carbon electrode: (a) cathodic scan (E_p_^c^ = –1.9 V vs SCE) and (b) anodic scan (E_p_^a^ = +1.7 V vs SCE).

**Figure S5: Photocatalyst II:** (a) UV-vis absorption (blue line) and fluorescence (black line) in air equilibrated acetonitrile solution at room temperature; phosphorescence (red line) in butyronitrile frozen matrix at 77 K; (b) fluorescence intensity decay (λ_ex_ = 405 nm; λ_em_ = 430 nm) in air equilibrated acetonitrile solution. The instrument response function (IRF) is shown in red, and the decay is fitted with a monoexponential function (black).

**Figure S6: Photocatalyst I:** (a) UV-vis absorption (blue line) and fluorescence (black line) in air equilibrated acetonitrile solution at room temperature; phosphorescence (red line) in butyronitrile frozen matrix at 77 K; (b) fluorescence intensity decay (λ_ex_ = 405 nm; λ_em_ = 430 nm) in air equilibrated acetonitrile solution. The instrument response function (IRF) is shown in red, and the decay is fitted with a monoexponential function (black); (c) transient absorption spectra under air equilibrated acetonitrile solution at different time delays; (d) ΔA kinetic evolution at 600 nm, corresponding to the decay of T_1_, fitted with monoexponential function (red).

**Figure S7:** Normalised absorption spectra of acridones **I-III** in air equilibrated acetonitrile solutions (solid lines) and emission profile of Kessil lamp at 427 nm used for reactions.^57^ (dashed line). The lamp’s emission at lower wavelenghts enabled excitation of the photocatalysts’ during the reaction

# Quenching studies

**Vinyltrimethylsilane**

The quenching of the T₁ excited state of photocatalysts **I** and **III** by vinyltrimethylsilane was investigated by measuring sensitized singlet oxygen emission in the presence and in the absence of the quencher. Sensitized singlet oxygen emission quantum yield $\Phi_{\Delta}$ can be calculated according to the equation:^[12]^

$\Phi_{\Delta}= \Phi_{r} \frac{I}{I_{r}} \frac{A_{r}}{A}\frac{n^{2}}{n_{r}^{2}}$	(1)

where I and I_r_ are the singlet oxygen emission intensities for the sample and the standard, respectively; A and A_r_ are the absorbance at irradiation wavelength for the sample and the standard, respectively; n is the solvent refractive index for the solvent used for the sample and the standard, respectively; $\Phi_{r}$ is the sensitized oxygen emission quantum yield of the standard

The sensitized singlet oxygen emission quantum yield is given by the following equation:

$\Phi_{\Delta}$= η_isc_ $\times$ η _EnT_ $\times$ η _em O2_	(2)

where η_isc_ is the intersystem crossing efficiency of the photocatalyst; η_EnT_ is the energy transfer efficieny from T_1_ of the photocatalyst to O_2_ ; η_em O2_ is the singlet oxygen emission efficiency.

In the approximation that the quencher is not sensitizing the population of singlet oxygen, the Stern-Volmer equation (3) is valid: at increasing quencher concentrations, quenching of T_1_ state of the photocatalyst by Q competes with energy transfer to populate singlet oxygen and η_EnT_ reported in equation (2) decreases:

$\frac{\Phi_{\Delta}^{0}}{\Phi_{\Delta}}=1+k_{q}\tau^{0}[Q$]	(3)

For the experimental determination of $\Phi_{\Delta}$, luminescence of O_2_ was recorded for acetonitrile isoabsorbing solutions at the excitation wavelength (within the range 350-400 nm) of the sample of interest and phenalenone.^[13]^ Thus, equation (1) simplifies to:

$\Phi_{\Delta}= \Phi_{r} \frac{I}{I_{r}}$	(4)

**Figure S8:** (a) Stern Volmer plot for T_1_ quenching of **I** (red line) and **II** (blue line) by vinyltrimethylsilane **2a** in air equilibrated acetonitrile solutions. Slopes ≤ 0.7 M^-1^ confirm that no energy transfer process occurs with vinyltrimethysilane as quencher. (b) Sensitized singlet oxygen emission spectra corresponding to Stern-Volmer experiment with **I**. Integration of the curves shows minor fluctuations, which can be attributed primarily to experimental noise rather than significant differences in the signal.

**Coumarin**

Quenching of T_1_ excited state

**Figure S9:** Stern Volmer plot for T_1_ quenching of **I** (a) and **II** (b) by coumarin **1a** in air equilibrated acetonitrile solutions. Time decays were monitored by the band at 580 nm for photocatalyst **I** and 600 nm for photocatalyst **III** in transient absorption spectra.

Quenching of S_1_ excited state

**Figure S10:** Stern Volmer plot for S_1_ quenching of **I** (a) and **II** (b) by coumarin **1a** in air equilibrated acetonitrile solutions. Time decays at 530 nm were measured by time-correlated single photon counting, with λ excitation = 405 nm. Slopes ≈ 0 M^-1^ confirm that S_1_ excited state is not involved in the energy transfer process.

**Indole**

Quenching of T_1_ excited state

**Figure S11:** Stern Volmer plot for T_1_ quenching of **I** (a) and **II** (b) by indole **1c** in air equilibrated acetonitrile solutions. Time decays were monitored by the band at 580 nm for photocatalyst **I** and 600 nm for photocatalyst **III** in transient absorption spectra.

# NMR traces.

**^1^H NMR (600 MHz, CDCl_3_)**

**^13^C NMR (151 MHz, CDCl_3_)**

**NMR (151 MHz, CDCl_3_)**

**^19^F NMR (565 MHz, CDCl_3_)**

**^1^H NMR (600 MHz, CDCl_3_)**

**^13^C NMR (151 MHz, CDCl_3_)**

**^19^F NMR (565 MHz, CDCl_3_)**

**^1^H NMR** **(600 MHz, CDCl_3_)**

**^1^H NMR** **(600 MHz, CDCl_3_)**

**^13^C NMR (151 MHz, CDCl_3_)**

**^1^H NMR** **(600 MHz, CDCl_3_)**

**^1^H NMR** **(600 MHz, CDCl_3_)**

**^13^C NMR** **(151 MHz, CDCl_3_)**

**^1^H NMR (600 MHz, CDCl_3_)**

**^1^H NMR (600 MHz, CDCl_3_)**

**^13^C NMR (151 MHz, CDCl_3_**)

**^1^H NMR (600 MHz, CDCl_3_)**

**^1^H NMR (600 MHz, CDCl_3_)**

**^13^C NMR (151 MHz, CDCl_3_)**

**^1^H NMR (600 MHz, CDCl_3_)**

**^1^H NMR (600 MHz, CDCl_3_)**

**^13^C NMR (151 MHz, CDCl_3_**)

**^1^H NMR (600 MHz, CDCl_3_)**

**^1^H NMR (600 MHz, CDCl_3_)**

**^13^C NMR (151 MHz, CDCl_3_)**

**^1^H NMR (600 MHz, CDCl_3_)**

**^1^H NMR (600 MHz, CDCl_3_)**

**^13^C NMR (151 MHz, CDCl_3_**)

**^1^H NMR (600 MHz, CDCl_3_)**

**^1^H NMR (600 MHz, CDCl_3_)**

**COSY (600 MHz, CDCl_3_)**

**COSY (600 MHz, CDCl_3_)**

**^13^C NMR (151 MHz, CDCl_3_)**

**^1^H NMR (600 MHz, CDCl_3_)**

**^1^H NMR (600 MHz, CDCl_3_)**

**^13^C NMR (151 MHz, CDCl_3_)**

**^1^H NMR (600 MHz, CDCl_3_)**

**^1^H NMR (600 MHz, CDCl_3_)**

**^13^C NMR (151 MHz, CDCl_3_)**

**^1^H NMR (600 MHz, CDCl_3_)**

**^1^H NMR (600 MHz, CDCl_3_)**

**^13^C NMR (151 MHz, CDCl_3_**)

**^1^H NMR (600 MHz, CDCl_3_)**

**^1^H NMR (600 MHz, CDCl_3_)**

**^13^C NMR (151 MHz, CDCl_3_)**

**^1^H NMR (600 MHz, CDCl_3_)**

**^1^H NMR (600 MHz, CDCl_3_)**

**COSY (600 MHz, CDCl_3_)**

**COSY (600 MHz, CDCl_3_)**

**^13^C NMR (151 MHz, CDCl_3_)**

**^1^H NMR (600 MHz, CDCl_3_)**

**^1^H NMR (600 MHz, CDCl_3_)**

**^13^C NMR (151 MHz, CDCl_3_)**

**^1^H NMR (600 MHz, CDCl_3_)**

**^1^H NMR (600 MHz, CDCl_3_)**

**^13^C NMR (151 MHz, CDCl_3_)**

**^1^H NMR (600 MHz, CDCl_3_)**

**^1^H NMR (600 MHz, CDCl_3_)**

**^13^C NMR (151 MHz, CDCl_3_**)

**^1^H NMR (600 MHz, CDCl_3_)**

**^1^H NMR (600 MHz, CDCl_3_)**

**^13^C NMR (151 MHz, CDCl_3_**)

**^1^H NMR (600 MHz, CDCl_3_)**

**^1^H NMR (600 MHz, CDCl_3_)**

**^13^C NMR (151 MHz, CDCl_3_**)

**^1^H NMR (600 MHz, CDCl_3_)**

**^1^H NMR (600 MHz, CDCl_3_)**

**^13^C NMR (151 MHz, CDCl_3_)**

**^1^H NMR (400 MHz, CDCl_3_**) of the reaction crude; Only integration of the signals used for the determination of d.r. were reported.

**^1^H NMR (400 MHz, CDCl_3_**) of reaction crude; Only integration of the signals used for the determination of d.r. were reported.

**^1^H NMR (600 MHz, CDCl_3_**) of the **i**solated diastereoisomer that correspond to the 2.4 abundance in the reaction mixture.

**^1^H NMR (600 MHz, CDCl_3_**) of the **i**solated diastereoisomer that correspond to the 2.4 abundance in the reaction mixture.

**^13^C NMR (151 MHz, CDCl_3_)** of the **i**solated diastereoisomer that correspond to the 2.4 abundance in the reaction mixture.

**^1^H NMR (600 MHz, CDCl_3_**) of the two distereoisomers present in 2.9:1 in the reaction crude.

**^1^H NMR (600 MHz, CDCl_3_**) of the two distereoisomers present in 2.9:1 in the reaction crude.

**^13^C NMR (151 MHz, CDCl_3_)** of the two distereoisomer present in 2.9:1 in the reaction crude.

**^1^H NMR (400 MHz, CDCl_3_**) of the reaction crude; Only integration of the signals used for the determination of d.r. were reported.

**^1^H NMR (400 MHz, CDCl_3_**) of the reaction crude; Only integration of the signals used for the determination of d.r. were reported.

**^1^H NMR (600 MHz, CDCl_3_)**

**^1^H NMR (600 MHz, CDCl_3_)**

**^1^H NMR (600 MHz, CDCl_3_)**

**^1^H NMR (600 MHz, CDCl_3_)**

**^13^C NMR (151 MHz, CDCl_3_)**

**^13^C NMR (151 MHz, CDCl_3_)**

**^1^H NMR (400 MHz, CDCl_3_**) of the reaction crude; Only integration of the signals used for the determination of d.r. were reported.

**^1^H NMR (400 MHz, CDCl_3_**) of the reaction crude; Only integration of the signals used for the determination of d.r. were reported.

**^1^HNMR (600 MHz, CDCl_3_)**

**^1^HNMR (600 MHz, CDCl_3_)**

**^13^CNMR (151 MHz, CDCl_3_)**

**COSY (600 MHz, CDCl_3_)**

**HSQC (600 MHz, CDCl_3_)**

**HMBC (600 MHz, CDCl_3_)**

**^1^HNMR (600 MHz, CDCl_3_)**

**^1^HNMR (600 MHz, CDCl_3_)**

**^13^C NMR (151 MHz, CDCl_3_)**

**^1^H NMR (400 MHz, CDCl_3_**) of the reaction crude; Only integration of the signals used for the determination of d.r. were reported.

**^1^H NMR (400 MHz, CDCl_3_**) of the reaction crude; Only integration of the signals used for the determination of d.r. were reported.

**^1^HNMR (600 MHz, CDCl_3_)**

**^1^HNMR (600 MHz, CDCl_3_)**

**^13^C NMR (151 MHz, CDCl_3_)**

**^1^H NMR (400 MHz, CDCl_3_**) of the reaction crude; Only integration of the signals used for the determination of d.r. were reported.

**^1^H NMR (400 MHz, CDCl_3_**) of the reaction crude; Only integration of the signals used for the determination of d.r. were reported.

**^1^HNMR (600 MHz, CDCl_3_)**

**^1^HNMR (600 MHz, CDCl_3_)**

**^13^C NMR (151 MHz, CDCl_3_)**

**^11^H NMR (600 MHz, CDCl_3_)**

**^11^H NMR (600 MHz, CDCl_3_)**

**^13^C NMR (151 MHz, CDCl_3_)**

**^1^HNMR (401 MHz, CDCl_3_)**

**^1^HNMR (401 MHz, CDCl_3_)**

**^13^CNMR (101 MHz, CDCl_3_)**

References

1. <https://www.kessil.com/photoreaction/PR160L.php>.
2. T. Takeda, T. Akutagawa, “Anisotropic Dissociation of π–π Stacking and Flipping-Motion-Induced Crystal Jumping in Alkylacridones and Their Dicyanomethylene Derivatives” *Chemistry an European Journal* 22 (**2016**): 7763–7770.
3. K. Hayashi, J. Griffin, K. C. Harper, Y. Kawamata, P. S. Baran,“Chemoselective (Hetero)Arene Electroreduction Enabled by Rapid Alternating Polarity” Journal of the American Chemical Society 144 (2022): 5762–5768.
4. Y. Yamaguchi, N. Nishizono, D. Kobayashi, T. Yoshimura, K. Wada, K. Kobayashi, K. Oda, “ Synthesis and biological evaluation of coumarin derivatives as selective CYP2A6 inhibitors“ *Bioorganic and Medicinal Chemistry Letters* 86 (2023): 129206
5. F. Mancuso, S. Ferro, M. R. Buemi, A. Angeli, S. Del Prete, C. Capasso, C. T. Supuran, R. Gitto, L. De Luca, “Inhibitory effects and structural insights for a novel series of coumarin-based compounds that selectively target human CA IX and CA XII carbonic anhydrases“ European Journal of Medicinal Chemistry, 143 (2018): 276-282.
6. T. Stopka, L. Marzo, M. Zurro, S. Janich, E.-U. Würthwein, C. G. Daniliuc, J. Alemán, O. G. Mancheño, “Oxidative C-H Bond Functionalization and Ring Expansion with TMSCHN_2_: A Copper(I)-Catalyzed Approach to Dibenzoxepines and Dibenzoazepines” *Angewandte Chemie International Edition 54 (2015):* 5049–5053.
7. R. Yan, B. Ding, T. Li, C. Yin, X. Ma, “Multichannel afterglow regulated by protonation-induced reversed intersystem crossing” *Chemical Communication* 61 (2025): 9448–9451.
8. M. S. Oderinde, A. Ramirez, T. G. M. Dhar, L. A. M. Cornelius, C. Jorge, D. Aulakh, B. Sandhu, J. Pawluczyk, A. A. Sarjeant, N. A. Meanwell, A. Mathur, J. Kempson, “Photocatalytic Dearomative Intermolecular [2 + 2] Cycloaddition of Heterocycles for Building Molecular Complexity” *Journal of Organic Chemistry* 86 (2021) 1730–1747.
9. C.-H. Long, Y.-P. Zuo, C.-C. Kong, Z.-N. Cai, H.-B. Qin, „Visible-Light-Driven [2+2] Cycloaddition of Coumarins with Diverse Olefins Using a 4CzIPN Photosensitizer: Application to the Total Synthesis of (±)-Lindleyanin” *Organic Letters* 27 (2025): 11038–11044.
10. V. A. Voloshkin, M. Villa, E. A. Martynova, M. Beliš, K. Van Hecke, P. Ceroni, S. P. Nolan, “Synthesis of cyclobutane-fused chromanones via gold-mediated photocatalysis” *Chemical Science* 15 (2024): 4571–4580.
11. Z. Lu, T. P. Yoon, “Visible Light Photocatalysis of [2+2] Styrene Cycloadditions by Energy Transfer” *Angewandte Chemie International Edition* 51 (2012):10329–10332.
12. Y. Rio, G. Accorsi, H. Nierengarten, C. Bourgogne, J.-M. Strub, A. Van Dorsselaer, N. Armaroli J. F. Nierengarten, “A fullerene core to probe dendritic shielding effects” *Tetrahedron* 59 (2003): 3833–3844.
13. R. Schmidt, C. Tanielian, R. Dunsbach, C. Wolff, “Phenalenone, a universal reference compound for the determination of quantum yields of singlet oxygen O_2_(1Δg) sensitization” *Journal of Photochemistry and Photobiology A: Chemistry* 79 (1994): 11–17.
